# Martinostat as a novel HDAC inhibitor to overcome tyrosine kinase inhibitor resistance in chronic myeloid leukemia

**DOI:** 10.1186/s13148-025-01921-0

**Published:** 2025-07-16

**Authors:** Haeun Yang, Vladimir Li, Su Jung Park, Sang Won Cheon, Anne Lorant, Aloran Mazumder, Jin Young Lee, Barbora Orlikova-Boyer, Claudia Cerella, Christo Christov, Gilbert Kirsch, Dag Erlend Olberg, Guy Bormans, Hyoung Jin Kang, Byung Woo Han, Michael Schnekenburger, Marc Diederich

**Affiliations:** 1 Research Institute of Pharmaceutical Sciences and Natural Products Research Institute, College of Pharmacy, Seoul National University, Seoul, 08826 Republic of Korea; 2 Laboratoire de Biologie Moléculaire et Cellulaire du Cancer, 1210 Luxembourg, Luxembourg; 3 Service d’Histologie, Faculté de Médicine, Université de Lorraine, INSERM U1256 NGERE, 54000 Nancy, France; 4 UMR CNRS 7053 LC2M, University of Lorraine, 57070 Metz, France; 5 Norsk Medisinsk Syklotronsenter AS, Postboks 4950, 0424 Nydalen, Oslo, Norway; 6 School of Pharmacy, University of Oslo, Oslo, Norway; 7 Laboratory for Radiopharmaceutical Research, Department of Pharmaceutical and Pharmacological Sciences, KU Leuven, Louvain, Belgium; 8 Department of Pediatrics, Seoul National University College of Medicine, Seoul National University Cancer Research Institute, Seoul National University Children’s Hospital, Seoul, 03080 Republic of Korea; 9 Present Address: Luxembourg Centre for Systems Biomedicine, Bioinformatics Core, Roudeneck, 1, Boulevard du Jazz, 4370 Esch, Luxembourg; 10 Present Address: Aging and Cancer Immuno-Oncology, Sanford Burnham Prebys Medical Discovery Institute, La Jolla, CA 92037 USA; 11 Present Address: Department of Biotechnology, Keimyung University, 1095 Dalgubeol-Daero, Dalseo-Gu, Daegu, 42601 Republic of Korea; 12 Present Address: Department of Cancer Research, Luxembourg Institute of Health (LIH), BAM Pavillon 2, 6 A Rue Nicolas-Ernest Barblé, 1210 Luxembourg, Luxembourg

**Keywords:** Chronic myeloid leukemia, HDAC inhibitors, Imatinib resistance, Cell death, Combination treatment

## Abstract

**Background:**

Chronic myeloid leukemia (CML) remains a therapeutic challenge, particularly in patients who develop resistance to standard tyrosine kinase inhibitors (TKIs) such as imatinib. Here, we present the first demonstration of the potent anti-leukemic activity of the histone deacetylase (HDAC) inhibitor martinostat in both TKI-sensitive and TKI-resistant CML.

**Methods and results:**

Structural and biochemical analyses confirmed the efficient and selective binding of martinostat to HDAC isoenzyme ligand-binding pockets, resulting in histone and tubulin hyperacetylation in both imatinib-sensitive and resistant CML cells, outperforming vorinostat, a clinically used HDAC inhibitor (HDACi). It selectively impaired CML cell proliferation and viability and induced apoptosis across various CML models, including resistant cell models and patient blasts, with minimal toxicity to healthy cells and low developmental toxicity in zebrafish. In addition to its single-agent efficacy, martinostat demonstrated enhanced anticancer effects when combined with imatinib, both in vitro and in vivo, significantly reducing tumor growth in resistant CML xenograft models. Mechanistically, mRNA-seq data showed that martinostat disrupted key survival signaling pathways and amplified apoptotic responses, contributing to its anticancer activity.

**Conclusions:**

These findings highlight the potential of martinostat as a selective, low-toxicity HDACi that, combined with TKIs, could provide an effective strategy to overcome drug resistance in CML and improve therapeutic outcomes.

**Supplementary Information:**

The online version contains supplementary material available at 10.1186/s13148-025-01921-0.

## Introduction

Chronic myeloid leukemia (CML) comprises approximately 15% of adult leukemia diagnoses and is characterized by a reciprocal translocation *t*(9;22)(q34;q11.2) involving the Abelson murine leukemia viral oncogene homolog (ABL)1 and breakpoint cluster region (BCR) genes, resulting in the Philadelphia chromosome and BCR-ABL oncoprotein, which drives proliferation and survival [1, 2]. Imatinib mesylate, the first FDA-approved tyrosine kinase inhibitor (TKI), targets the BCR-ABL ATP-binding site and inhibits phosphorylation-dependent signaling [3]. Additional TKIs (dasatinib, bosutinib, and nilotinib) have further improved outcomes, decreasing annual CML mortality from 10 to 20% to 1–2%, and increasing the 5-year relative survival from 22% in the mid-1970s to 70% [1, 4]. Nonetheless, TKI side effects and resistance to TKIs underscore the need for novel therapeutic strategies.

The modulation of epigenetic mechanisms plays an important role in cancer progression [5] and CML [6, 7]. Histone deacetylases (HDACs) remove acetyl groups from lysine residues on histone tails, leading to chromatin condensation and repression of genes related to survival, cell cycle control, and programmed cell death [8]. HDACs are broadly involved in multilineage development and the formation of transcriptional complexes during hematopoiesis. In normal hematopoiesis, they regulate the formation of transcriptional complexes involving HDACs and transcription factors, leading to the modulation of hematopoiesis-related gene expression [9].

Overexpression of HDAC1, 2, and 3 has been observed in leukemia stem cells (LSCs) from patients with CML [10], and TKI treatment significantly reduced HDAC6 mRNA levels [2].

Several HDAC inhibitors (HDACi) have been used in clinical trials involving patients with CML [NCT00451035, NCT00015925]. Furthermore, HDACi synergize with TKIs in vitro [11, 12], and clinical trials have investigated the combinatorial effect of HDACi with TKIs [NCT00816283, NCT00686218]. Pan-HDACi suberoylanilide hydroxamic acid (SAHA, vorinostat) was the first FDA-approved HDACi for treating cutaneous T-cell lymphoma [13]. In CML, SAHA synergistically enhances apoptotic cell death in combination with dasatinib [14].

The HDACi martinostat [15] showed improved brain penetration. ^11^C-tagged martinostat has been used in positron emission tomography (PET) brain neuroimaging studies of schizophrenia [16] and Alzheimer’s [17]. However, its anticancer potential as an HDACi has not yet been tested in leukemia.

**Fig. 1:**
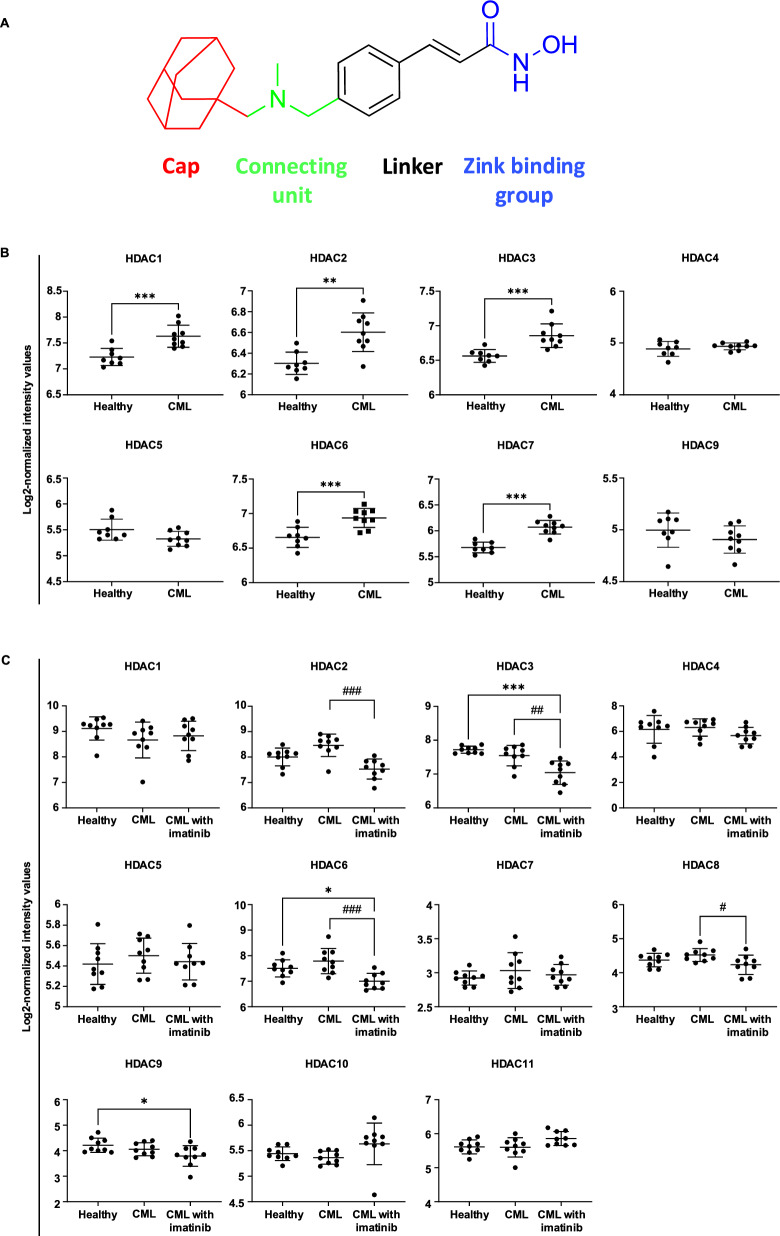
Structure of martinostat and computational analysis of publicly available GEO datasets. **A** Structure of HDAC inhibitor martinostat. **B**, **C** Gene expression level of HDACs in the CML samples compared to healthy controls from the GSE5550 dataset (**B**), and before and after imatinib therapy from the GSE33075 dataset (**C**). Data are presented as the mean ± SD. *P* values were determined by unpaired two-tailed t-test (**B**) and one-way ANOVA followed by Šídák’s multiple comparisons test (**C**). *, **, and *** indicate *P* < 0.05, *P* < 0.01, and *P* < 0.001, respectively, versus the healthy group. # and ### indicate *P* < 0.05 and *P* < 0.001, respectively, versus the CML group treated with imatinib

We validated the anti-leukemic effect of martinostat (Fig. 1A) against CML by using resistant cell models and patient blasts. Martinostat demonstrated drug-like properties in line with Lipinski’s rule of five. It increased histone acetylation at concentrations lower than the clinically used HDACi SAHA. Compared to SAHA, martinostat exhibited greater potency in inhibiting colony formation, inducing morphological changes, and modulating the cell cycle phase distribution and proliferation. Notably, martinostat demonstrated improved selectivity for CML cells compared to healthy cells. In addition, martinostat showed a synergistic cytotoxic effect with imatinib in both imatinib-sensitive and resistant K562 cells by reducing viability and proliferation, eventually leading to caspase-dependent cell death and inhibition of the BCR-ABL signal transducer and activator of transcription 5 (STAT5) pathway. Additionally, the enhanced therapeutic potential of the combination of martinostat and imatinib has been validated in vivo using xenograft assays. These findings indicate that martinostat is a promising preclinical drug candidate for treating imatinib-sensitive and resistant CML.

## Material and methods

### Public data and computational analyses

GSE5550 [18] and GSE33075 [19] public patient datasets were retrieved from the Gene Expression Omnibus (GEO) database (https://www.ncbi.nlm.nih.gov/geo/). GSE5550: bone marrow (BM) samples from 17 patients were sorted for CD34^+^ hematopoietic stem and progenitor cells, including eight samples from healthy donors and nine samples from untreated CML patients. Gene expression values were analyzed using gene ID from the gene platform GPL201. GSE33075: Nine samples from healthy donors and nine samples from patients diagnosed with CML. Gene expression values were analyzed using gene ID from the gene platform GPL570.

### In silico drug-likeness properties

The in silico drug-likeness properties of HDAC inhibitors were evaluated using the web-based programs Molinspiration Cheminformatics (http://www.molinspiration.com/) and PreADMET version 2.0 (https://preadmet.bmdrc.kr).

### Computational docking

Docking simulations were conducted using the crystal structures of HDACs deposited in the Protein Data Bank (PDB) under the codes 4BKX (HDAC1), 4LXZ (HDAC2), 4A69 (HDAC3), 2VQM (HDAC4), 5EDU (HDAC6), 3C0Z (HDAC7), 1T69 (HDAC8), and 7SGG (HDAC10). Protein structures were prepared using the Protein Preparation Workflow by deleting water molecules and the originally bound ligands [20]. Ligands, including SAHA and martinostat, were generated in various protonation states at pH 7.0, prepared using LigPrep in the Maestro suite by adding metal-binding states at the Optimized Potentials for Liquid Simulations Version 4 (OPLS4) force field [21]. Grids for docking simulations were generated using the Receptor Grid Generation tool in the Maestro suite, incorporating metal coordination at Zn^2^⁺, an essential component of the binding moiety. The center of the grid was designated at the location of the catalytic triad consisting of two histidine (His) and one aspartic acid (Asp) residues common to HDACs. Docking simulations were performed using Glide (Schrödinger, Inc., Seongnam-si, Korea) with the prepared HDACs and ligand structures [22].

### Prediction of binding affinity

Maestro’s Prime Molecular Mechanics-Generalized Born Surface Area (MM/GBSA) tool was used to calculate MM/GBSA and predict the binding affinities between HDACs and ligands (Schrödinger Release 2023-4: LigPrep, Schrödinger, LLC, New York, NY, 2023). The docking simulation results were energy-minimized using the minimization option in the calculation tool and the variable-solvent generalized born (VSGB) solvation model in the OPLS4 force field [23].

### Compounds

The compound martinostat was synthesized, as shown in Fig. S1. Imatinib and etoposide were purchased from Sigma-Aldrich (St. Louis, MO, USA). SAHA was purchased from Cayman Bio-Connect (Huissen, the Netherlands). 

### HDAC activity assays

HDAC activity assays were performed as previously reported [24]. The fluorescence signal was measured using a SpectraMax i3x fluorimeter (Molecular Devices Korea) with SoftMax Pro software at an excitation wavelength of 360 nm and an emission wavelength of 460 nm. The 50% inhibitory concentration (IC_50_) values for HDAC activity were determined using Prism 9.0 software (version 9.4.1; GraphPad, La Jolla, CA, USA), based on nonlinear regression methods.

### Cell culture

The human CML K562 cell line was purchased from Deutsche Sammlung für Mikroorganismen und Zellkulturen (DSZM, Braunschweig, Germany). The human CML KBM5 cell line was gifted by Dr. Bharat B. Aggarwal (MD Anderson Cancer Center, Houston, TX, USA). Imatinib-resistant KBM5-T315I cells (KBM5-IR) were established as previously described [25]. The K562-R cell line was a gift from Professor Dong-Wook Kim (Department of Hematology, Seoul St. Mary’s Hematology Hospital, The Catholic University of Korea, Seoul, Republic of Korea), and resistance mechanisms were previously described [26]. Normal human B-lymphocytic RPMI 1788 cell line was obtained from the Korean Cell Line Bank (Seoul, Republic of Korea). The cells were cultured at 37 °C in a humidified atmosphere with 5% CO_2_ in a medium containing 1% antibiotics (streptomycin and penicillin), antimycotics (Lonza, Walkersville, MD, USA), and 10% (20% for RPMI 1788 cells) heat-inactivated fetal bovine serum (FBS; Biowest, Riverside, MO, USA). CML K562 and normal RPMI 1788 cells were cultured in RPMI 1640 medium (Lonza, Basel, Switzerland). KBM5 cells were cultured in Iscove’s Modified Dulbecco’s medium (IMDM; Lonza). KBM5-IR and K562-R sublines were cultured in the same medium as their parental cell lines, supplemented with 1 µM and 0.8 µM imatinib, respectively. The cell lines were tested monthly to ensure they were free from mycoplasma contamination.

Peripheral blood mononuclear cells (PBMCs) from CML patients (Seoul National University Hospital, Seoul, Korea; Table S1) were isolated from buffy coats as previously described [11]. Isolated PBMCs were cultured in RPMI 1640 medium supplemented with 10% heat-inactivated fetal bovine serum (FBS). The cell lines were used within a passage range of 10–20.

### Cell proliferation and viability assays

Cell proliferation and viability were assessed using the trypan blue exclusion method (Lonza, Basel, Switzerland) as previously described [12]. The number of cells per milliliter was counted using a hemocytometer (Marienfeld, Lauda-Königshofen, Germany). The percentage of growth inhibition (GI) was calculated as previously reported [11]. The IC_50_ values for GI and cell death (GI_50_ and lethal dose [LD] _50_, respectively) were determined using Prism software (version 10.0) based on nonlinear regression methods.

### Zebrafish toxicity assay

Zebrafish embryos were treated with 0.003% phenylthiourea (Sigma-Aldrich) 14 h before the assay to inhibit pigmentation. Two hours before the assay, the chorion was removed from the embryos, which were then treated for up to 24 h with martinostat at the indicated concentrations in the 24-well plates. Viability and heartbeat rate were assessed using a light microscope (Carl Zeiss Stereo Microscope DV4, Seoul, Korea). Images were captured by fixing zebrafish embryos onto glass slides with 3% methylcellulose (Sigma-Aldrich). This study was performed in accordance with a protocol approved by the Institutional Animal Care and Use Committee of Seoul National University, Korea (SNU-191218-5-2).

### Protein extraction and western blots

To prepare whole-cell extracts, cells were harvested, washed in cold 1× phosphate-buffered saline (PBS), and lysed in Mammalian Protein Extraction Reagent (M-PER™, Thermo Fisher Scientific, MA, USA) supplemented with a protease inhibitor cocktail (Complete, EDTA-free, Roche, Basel, Switzerland) according to the manufacturer’s instructions. The protein concentration was measured using the Bradford assay. Proteins were aliquoted and stored at − 80 °C. Subsequently, the proteins were subjected to sodium dodecyl sulfate–polyacrylamide gel electrophoresis (SDS-PAGE) and transferred to PVDF membranes (GE Healthcare, Seoul, Korea). After the transfer, the membranes were blocked with PBS-Tween containing the appropriate blocking agent (5% milk or 5% BSA) for 1 h at room temperature. Membranes were incubated with the selected primary antibodies overnight at 4 °C (Table S2). After incubation with the primary antibodies, the membranes were washed with PBS-Tween 20 (PBS-T; Sigma-Aldrich, MO, USA). Membranes were then incubated with a species-appropriate HRP-conjugated secondary antibody (Santa Cruz Biotechnology, TX, USA) in PBS-T containing 5% milk for 1 h at room temperature. As it is technically challenging to strip very strong signals from histone H4 or α-tubulin from western blot membranes, we used histone H1 and β-actin as *bona fide* loading controls, as previously described [10, 12]. After incubation with the secondary antibody, membranes were washed with PBS-T. Chemiluminescence signals were detected using an ECL Plus Western Blotting Detection System (RPN2235; GE Healthcare, Little Chalfont, UK). Western blot quantification was done using an ImageQuant LAS 4000 mini system (GE Healthcare), and the corresponding fold-change values relative to the control after normalization to the respective loading controls are indicated under the immunoblot pictures.

### mRNA sequencing and data processing

Total RNA was isolated using the TRIzol™ reagent (Invitrogen, Seoul, Korea). RNA quality was assessed using an Agilent 2100 bioanalyzer (Agilent Technologies, Amstelveen, The Netherlands), and RNA quantification was performed using an ND-2000 Spectrophotometer (Thermo Inc., DE, USA). Libraries were prepared from total RNA using the NEBNext Ultra II Directional mRNA-Seq Kit (New England BioLabs Inc., Hitchin, UK). mRNA isolation was performed using the Poly(A) RNA Selection Kit (Lexogen, Inc., Vienna, Austria). The isolated mRNAs were used for cDNA synthesis and shearing following the manufacturer’s instructions. Indexing was performed using Illumina indexes 1–12. Enrichment was performed by PCR. Subsequently, libraries were checked using TapeStation HS D1000 ScreenTape (Agilent Technologies, Amstelveen, The Netherlands) to evaluate the mean fragment size. Quantification was performed using a library quantification kit and StepOne Real-Time PCR System (Life Technologies, Inc., CA, USA). High-throughput sequencing was performed as paired-end 100 sequencing using NovaSeq 6000 (Illumina, Inc., CA, USA).

AccuGene (Incheon, Korea) performed mRNA-sequencing analysis. Raw sequencing reads in FASTQ format were initially evaluated for quality using FastQC (version v0.12.1, Babraham Institute, UK). Low-quality bases and adapter sequences were removed using Trim Galore (version 0.6.10; Babraham Institute, UK) and Trimmomatic v.0.38 to ensure that clean reads were prepared for further analyses. Trimmed sequencing data were aligned to the reference human genome (hg38) using HISAT2 (version 2.2.1; Center for Computational Biology, Johns Hopkins University, MD, USA). The resulting SAM files were then converted to the BAM format and sorted using SAMtools (version 1.21, Wellcome Sanger Institute, UK). Gene-level quantification was conducted using featureCounts (version 1.5.3, University of South Carolina, USA), with GENCODE Release 38 as the reference annotation. Differential expression analysis was performed using the DESeq2 package in R, utilizing gene count data obtained from featureCounts. Genes with an adjusted *P* value < 0.05 were considered significantly differentially expressed. Gene ontology (GO) enrichment analysis was performed using the PANTHER classification tool (released 20,240,807, University of Southern California, USA) and clusterProfiler R package (v.4.16.0). KEGG pathway analysis was also carried out using clusterProfiler R package (v.4.16.0). Based on the P value, the top 20 enriched GO terms were further summarized and visualized using Revigo (University of California, San Francisco, CA, USA) and enrichplot package (v.1.28.2) of RStudio to minimize redundancy and provide a clear representation of biological functions.

Raw FASTQ files from K562 wild-type (WT) and K562-R cell lines were first assessed for quality using FastQC (v0.12.1) [27]. The files were trimmed for quality and adapter sequences using Trimmomatic v.0.38 [27]. Cleaned reads were aligned to the GRCh38 human reference genome (retrieved from Ensembl) using HISAT2 (v2.2.1), employing default parameters [28, 29]. Aligned reads were quantified at the gene level using featureCounts (v2.0.6), based on the corresponding GTF annotation file (retrieved from Ensembl) [29, 30]. The resulting raw count matrix was loaded into DESeq2 (v1.48.1) for normalization and differential gene expression analysis [31]. Genes with an adjusted P value (Benjamini–Hochberg correction) < 0.05 and |log_2_FC|> 2 were considered significantly differentially expressed [32].

### Colony formation assays

Cells were seeded at 10^3^ cells/mL in a semi-solid methylcellulose medium (MethoCult H4230; StemCell Technologies Inc., Vancouver, Canada) supplemented with 10% FBS in 12-well plates. After 10 days of incubation, colonies were detected by adding 1 mg/mL of 3-(4,5-dimethylthiazol-2-yl)−2,5-diphenyltetrazolium bromide (MTT) reagent (Sigma). Images were acquired using an ImageQuant LAS 4000 analyzer (GE Healthcare). Colonies were counted and analyzed using ImageJ software (version 1.53; National Institutes of Health, Bethesda, MD, USA).

### Cell cycle analysis

Cells were collected, washed with 1× PBS by centrifugation, and fixed with 70% ethanol for 30 min. After fixation, the cells were washed with 1× PBS by centrifugation and stained with 1 mg/ml propidium iodide (PI, Sigma-Aldrich, St. Louis, MO, USA) for 10 min. The cell cycle distribution was quantified using a FACS Lyric flow cytometer (BD Biosciences, CA, USA). The data were analyzed using FlowJo^®^ software (version 10.2.1; Treestar, Ashland, OR, USA).

### Measurement of high mobility group box 1 release

The cells were centrifuged, and the supernatant was collected. The high mobility group box (HMGB)1 levels in the supernatants were quantified using an HMGB1 ELISA kit according to the manufacturer’s instructions (Tecan, Männedorf, Switzerland).

### Cell death assessment

Phosphatidylserine exposure was quantified using an Annexin V-Fluorescein Isothiocyanate (FITC) Apoptosis Detection Kit I (BD Biosciences), according to the manufacturer’s protocol. The samples were analyzed using a FACSCalibur flow cytometer (BD Biosciences), and the data were processed using the FlowJo software (version 10.4.1; Treestar, Ashland, OR, USA). The combination index (CI) was calculated using CompuSyn software (ComboSyn Inc., Paramus, NJ, USA).

### Detection of intracellular ATP content

The intracellular ATP content was determined using a CellTiter-Glo^®^ Luminescent Cell Viability Assay (Promega, Madison, WI, USA). Cells were seeded at 2 × 10^5^ cells/mL in a 96-well plate, treated with the indicated concentrations of the compounds, and incubated for 24, 48, and 72 h. After incubation, the cell suspension was mixed with the CellTiter-Glo reagent and incubated for 10 min at room temperature. The results were quantified using a Centro LB 960 microplate reader (Berthold Technologies, Bad Wildbad, Germany).

### Caspase-3/7 activity assay

Caspase-3/7 activity was assessed using the Caspase-Glo^®^ 3/7 assay (Promega, WI, USA). The caspase-3/7 assay reagent was added to the samples at a 1:1 ratio, and the cells were incubated for 1 h at room temperature. The luminescence was measured using a Centro LB 960 microplate reader (Berthold Technologies, Bad Wildbad, Germany).

### Transmission electron microscopy

The cells were pelleted (5 × 10^6^) and fixed overnight in 2.5% glutaraldehyde (Electron Microscopy Sciences, PA, USA) diluted in 0.1 M sodium cacodylate buffer, pH 7.2 (Electron Microscopy Sciences). The cells were rinsed twice with sodium cacodylate buffer and post-fixed with 2% osmium tetroxide for 2 h at room temperature. The samples were washed with distilled water and stained with 0.5% uranyl acetate (Electron Microscopy Sciences, PA, USA) overnight at 4 °C. The samples were dehydrated using a graded series of ethanol solutions, followed by propylene oxide, and infiltrated with a 1:1 mixture of propylene oxide and Spurr’s resin. The samples were embedded in Spurr’s resin, mounted in molds, and polymerized in an oven at 56 °C for 48 h. Ultrathin sections (70–90 nm) were obtained using an EM UC7 ultramicrotome (Leica, Seoul, Korea). Sections were stained with uranyl acetate and lead citrate and observed under a JEM1010 transmission electron microscope (JEOL, Tokyo, Japan) or a Talos L120C transmission electron microscope (Thermo Fisher Scientific, MA, USA).

### Xenograft assays

Xenograft studies utilized 6-week-old female BALB/c nude mice (RAON Bio, Yongin, Korea). One day before inoculation with K562-R cells, whole-body irradiation was conducted at 2.5 Gy using an X-RAD iR160 irradiator. A total of 10^7^ K562-R cells were resuspended in 100 μL of a 1:1 mixture of 1× PBS and Geltrex™ (Thermo Fisher Scientific) and injected into the right flanks of the mice. The body weight and tumor size of the mice were measured every three days using a digital caliper (SD500-150PRO, Sincon, Korea). When the tumor volume reached approximately 100 mm^3^, the mice were randomly assigned to the vehicle, martinostat (80 mg/kg), imatinib (50 mg/kg), or martinostat plus imatinib co-treatment groups. Martinostat (80 mg/kg) and imatinib (50 mg/kg) were injected intraperitoneally in corn oil/10% DMSO every three days. The tumor volume was calculated using the following formula: (width)^2^ × 0.5. The experiment was terminated when the tumor size in the vehicle group reached 10% of body weight. Tumors were excised, fixed in 4% formalin, and stained with hematoxylin and eosin (H&E). The remaining tissues were stored in 4% formalin. For euthanasia, the mice were exposed to CO_2_ for 3 min of active exposure, followed by 10 min of passive exposure. For serum biochemical analyses, blood samples were collected from the hearts of each mouse on day 18. Animal experiments were performed according to protocols approved by the Institutional Animal Care and Use Committee of Seoul National University, Korea (SNU-220704-6-3).

### Determination of blood serum biochemical characteristics

Blood samples were centrifuged at 1500*g*. After 15 min, the upper serum layer was collected, and the levels of alanine aminotransferase (ALT), aspartate aminotransferase (AST), blood urea nitrogen (BUN), and creatinine (CRE) were determined using a Dri-Chem 3500i analyzer (Fujifilm, Seoul, Korea).

### Immunohistochemistry analysis

Tumor tissue sections of 5 µm thickness were embedded in paraffin, dried, and rehydrated using xylene and ethanol (Duksan Science, Seoul, Korea). Antigen retrieval was performed using a 10 mM sodium citrate buffer (pH 6.0) containing 0.05% Tween 20 and heated in a water bath at 98 °C for 20 min. Endogenous peroxidase activity was blocked with 30% hydrogen peroxide for 10 min. Slides were extensively washed with 1× TBS (Tris-Buffered Saline) and incubated overnight at 4 °C with primary antibodies, such as anti-Ki67 (ab16667, 1:200, Abcam), anti-acetylated histone H4 (06–866, 1:5000, Millipore), or anti-acetylated α-tubulin (sc-23950, 1:250, Santa Cruz Biotechnology, TX, USA). After overnight incubation, the sections were incubated with a secondary antibody (VECTASTAIN® ABC-HRP Kit, Peroxidase [Rabbit IgG], PK-4001, Vector Laboratories, CA, USA) for 30 min at room temperature. 3,3′-diaminobenzidine (DAB) staining was performed using the ImmPACT DAB Substrate Kit, Peroxidase (SK-4105, Vector Laboratories, CA, USA). Finally, the slides were counterstained with hematoxylin. Slide scanning was performed using a ZEISS Axioscan 7 slide scanner (ZEISS, Oberkochen, Germany).

### Statistical analyses

All animal experiments were randomized, and no animals were excluded from the analysis. All statistical analyses were performed using Prism software, except for specific bioinformatic studies. The significance threshold for *P* values was set at *P* < 0.001. Appropriate adjustments were made to address the multiple tests. The details of the statistical tests and *P* value adjustments are included in the figure legends.

### Ethical considerations

Ethical Compliance: All procedures performed in this study involving human participants followed the ethical standards of the institutional and/or national research committee and the 1964 Helsinki Declaration and its later amendments or comparable ethical standards. All animal experiments were performed according to the protocols approved by the Institutional Animal Care and Use Committee at Seoul National University, Korea: SNU-220704-6-3; zebrafish toxicity assay: SNU-191218-5-2. CML patient samples were used after obtaining approval from the Seoul National University Institutional Review Board (IRB No. E2410/002–001).

## Results

### Translational relevance of HDAC inhibition in CML

In the absence of publicly available TCGA datasets specific to CML, we first investigated the transcriptomic dataset GSE5550 to compare the gene expression profiles of healthy hematopoietic cells and CML BM stem cells (SCs). Our results showed that HDACs 1, 2, 3, 6, and 7 were significantly upregulated in CML BM SCs compared with their healthy counterparts (Fig. 1B). These findings indicated that CML BM SCs are dependent on HDAC class I and IIb enzymes.

We further explored the clinical relevance of HDACs as pharmacological targets in CML using the public gene expression dataset, GSE33075. We compared gene expression before and after eight days of imatinib treatment at therapeutically relevant concentrations. Our results demonstrate that HDACs 2, 6, and 8 were downregulated by imatinib treatment (Fig. 1C). Based on these findings, we hypothesized that multiple HDACs were essential for CML cell proliferation and survival. This prompted us to investigate the anti-CML activity of pan-HDACis in drug-sensitive and drug-resistant CML cell types, both alone and in combination with TKIs.

### In vitro HDAC inhibition by martinostat and in silico drug-likeness properties

Considering that HDAC class I and IIb enzymes are elevated and critical for CML cell survival, we next evaluated the in vitro inhibitory potency of martinostat and its drug-likeness properties to determine its suitability as a pan-HDAC inhibitor for CML treatment.

We conducted total HDAC activity assays to assess the effect of martinostat on the deacetylase activity in vitro. Martinostat inhibited the deacetylase activity (HDAC class I to IV) measured in nuclear extracts isolated from exponentially growing K562 cells with an IC_50_ of 9 nM compared to SAHA’s IC_50_ of 23 nM. We also analyzed the effect of martinostat on HDAC isoenzyme activity and compared it to that of SAHA. Our results indicate that martinostat significantly reduced the activities of HDAC2, HDAC6, and HDAC10 at lower concentrations than SAHA (Table 1). Overall, martinostat exhibited an inhibitory effect on HDAC isoenzymes, similar to that of SAHA, further confirming its pan-HDAC inhibition potential, in contrast to previous reports suggesting that martinostat acts as a highly specific HDAC2 inhibitor.

**Table 1:** IC_50_ values for martinostat and SAHA against total and selected HDAC activities

HDAC		IC_50_ (nM)^1^		Ratio IC_50_ (SAHA/martinostat)
Class	Member	Martinostat	SAHA	
All	NA	9 ± 2	23 ± 6	2.6 ± 0.3**
I	HDAC1	80 ± 3	89 ± 6	1.1 ± 0.1
	HDAC2	61 ± 7	91 ± 7	1.5 ± 0.1**
	HDAC3	79 ± 8	77 ± 5	1.0 ± 0.1
	HDAC8	1647 ± 155	1155 ± 95	0.7 ± 0.1**
IIb	HDAC6	70 ± 7	98 ± 5	1.4 ± 0.1**
	HDAC10	72 ± 7	122 ± 11	1.7 ± 0.1**
IV	HDAC11	34 ± 1	41 ± 7	1.2 ± 0.2

^1^IC_50_: Drug concentration inhibiting 50% of the specified histone deacetylase (HDAC) activity. IC_50_ values were calculated from the inhibitory dose–response curves using the negative solvent control DMSO set to 100% activity. Data are presented as mean ± SD of at least three independent experiments. P values were calculated using an unpaired two-tailed t-test with *P ≤ 0.05, **P ≤ 0.01. NA, non-applicable; SAHA, suberoylanilide hydroxamic acid

Furthermore, we investigated the in silico drug-likeness properties of martinostat and the FDA-approved HDAC inhibitors, SAHA, Panobinostat, and trichostatin A. The results demonstrated that martinostat satisfied Lipinski’s Rule of Five and exhibited drug-like characteristics comparable to those of other FDA-approved HDAC inhibitors (Table 2).

**Table 2:** In silico prediction for the drug-likeness properties of HDAC inhibitors

Method	Parameter	Theoretical values	Martinostat	SAHA	Panobinostat	Trichostatin A
Lipinski’s rule of five	MW (Da)	$\le$ 500	354	264	349	302
	Number of atoms	20 ≤ × ≤ 70	26	19	26	22
	Volume (Å^3^)	NA	349	256	330	293
	miLogP	$\le$ 5	3.87	2.47	2.31	2.68
	Hydrogen bond donors	$\le$ 5	2	3	4	2
	Hydrogen bond acceptors	$\le$ 10	4	5	3	3
	Molar refractivity	NA	106.87	72.85	103.76	87.28
Veber’s rule	Rotatable bonds	$\le$ 10	6	8	8	6
	TPSA (Å^2^)	$\le$ 140	52.56	78.42	77.14	69.64
Absorption	IA (%)	$\ge$ 70	93.47	84.30	89.23	92.66
	PPB (%)	< 90	78.67	64.70	78.3	83.38
	BBBP	0.1 $\le$ MA $\le$ 2	2.42	0.19	1.16	0.46
Toxicity	Mouse	NA	Negative	Positive	Negative	Negative

BBBP, blood–brain barrier penetration; IA, intestinal absorption; MA, middle absorption; miLogP, octanol–water partition coefficient; MW, molecular weight; NA, not applicable; PBB, plasma protein binding; TPSA, topological polar surface area

### Martinostat efficiently binds to the ligand-binding pocket of HDAC isoenzymes

**Fig. 2:**
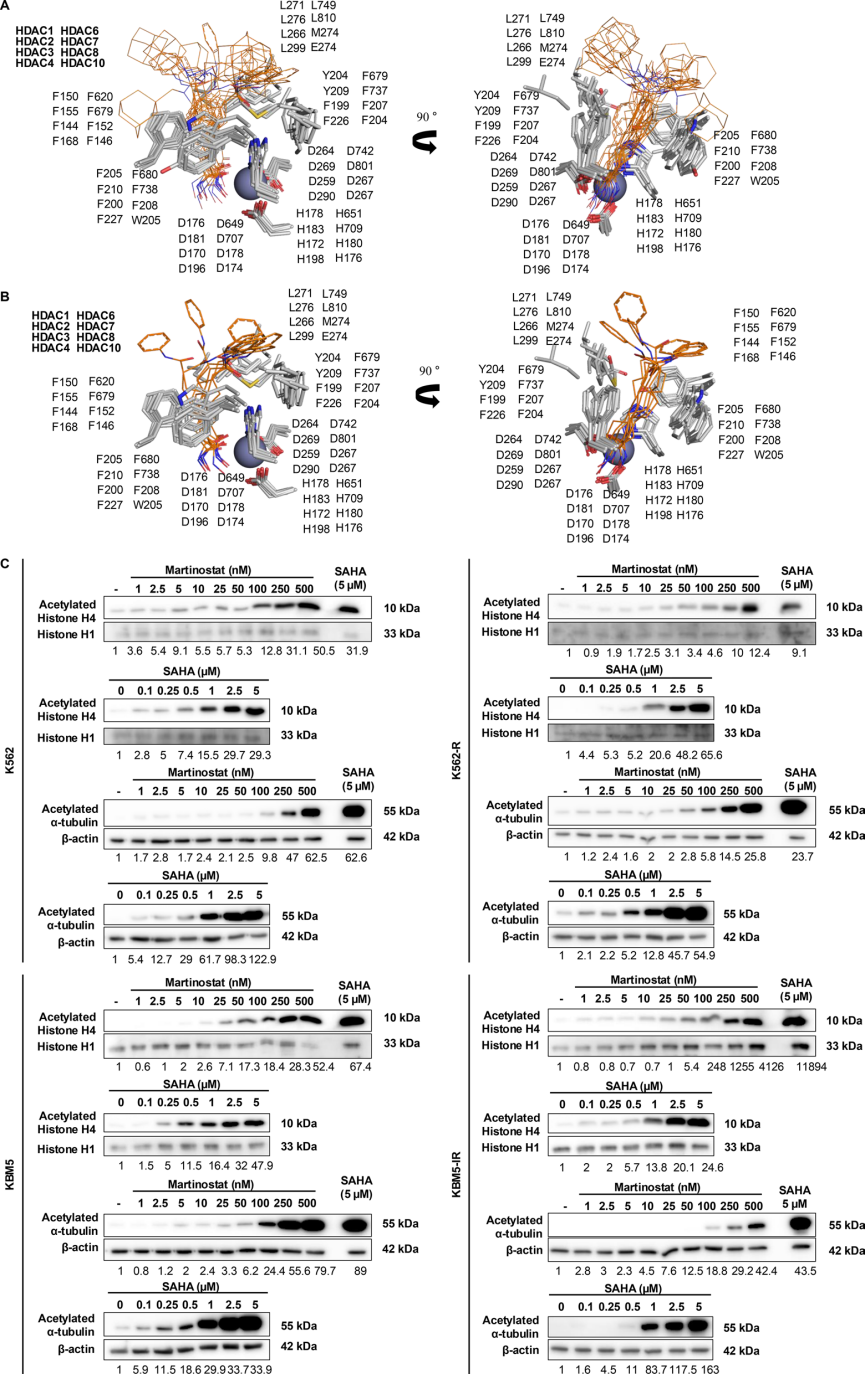
Martinostat Inhibits HDAC Activities In Vitro. **A**, **B** Residue comparison of HDAC isoenzymes via alignment, focusing on the catalytic triad with Zn^2^⁺ ion and residues involved in hydrophobic interactions with martinostat (**A**) and SAHA (**B**). Numbered residues forming hydrophobic interactions in the binding sites correspond to HDAC1 to HDAC11 from top to bottom. The zinc atom is shown as a purple sphere. **C** Acetylation levels of histone H4 and α-tubulin in the indicated CML cell lines after 24 h of treatment with increasing concentrations of martinostat and SAHA. β-actin and histone H1 were loading controls for α-tubulin and histone H4, respectively. Immunoblot images show a representative result from three independent experiments; the quantifications below each blot correspond to the depicted representative blot

Having confirmed the pan-HDAC inhibition potential and favorable drug-likeness profile of martinostat, we investigated the structural basis of its activity by conducting computational docking studies against multiple HDAC isoenzymes. Docking simulations of martinostat (Fig. 2A) and SAHA (Fig. 2B) with HDACs showed that both compounds interacted with the binding pocket through similar moieties, as evidenced by their alignment.

Analysis of the ligand-binding pocket indicated that Zn^2+^ binds to a catalytic triad comprising two histidine residues and one aspartic acid residue. At the entrance of the ligand-binding pocket, the phenylalanine and tryptophan residues were aligned in parallel, creating a hydrophobic region. This arrangement was consistently observed in both the crystal structures used for docking simulations and simulation results, where these residues interacted with the hydrophobic linkers of the ligands via *π*-interactions (Fig. 2A, B).

By comparing the docking simulation results, we confirmed that the HDACs and their ligands bind in comparable forms. Both martinostat and SAHA bind to Zn^2^⁺ in the catalytic domain of all HDACs, with their linkers aligned in parallel through π-interactions with phenylalanine and tryptophan residues (Fig. S2A, B). After comparing the overall structural differences, we calculated quantitative binding affinities. Calculations from docking simulations showed that martinostat had higher binding affinities than SAHA for HDAC1, HDAC2, HDAC6, and HDAC10 (Table 3), which was in line with the HDAC inhibition results (Table 1).

**Table 3:** In silico docking affinity scores of martinostat and SAHA against selected HDACs

HDAC			Binding affinity score (kcal/mol)^a^	
Class	Member	PDB code	Martinostat	SAHA
I	HDAC1	4BKX	− 15.83	− 15.46
	HDAC2	4LXZ	− 15.51	− 14.52
	HDAC3	4A69	− 15.24	− 15.45
	HDAC8	1T69	− 5.62	− 8.11
IIa	HDAC4	2VQM	− 15.50	− 15.45
	HDAC7	3C0Z	− 13.26	− 4.35
IIb	HDAC6	5EDU	− 18.07	− 10.89
	HDAC10	7SGG	− 14.93	− 4.02

^a^Binding affinity energy values (kcal/mol) for the indicated Protein Data Bank (PDB) codes were calculated using the Schrödinger program. SAHA was used as a pan-HDACi reference

Quantitative docking binding affinity results demonstrated that martinostat displayed more potent binding affinities than SAHA for the tested HDACs, suggesting a moderately different HDAC inhibitory profile for the two compounds. The binding affinity energy values were similar for certain HDACs and distinct from the others (Table 3). Altogether, our results confirm that martinostat, similar to SAHA, binds with high affinity to class I and IIb HDACs, reinforcing its pan-HDAC inhibitory potential.

### Martinostat leads to histone and tubulin hyperacetylation in TKI-sensitive and -resistant CML cell types

After establishing the binding of martinostat within the HDAC catalytic pocket, we examined whether this translates into effective inhibition of key HDAC substrates in TKI-sensitive and TKI-resistant CML cells. We compared the effects of martinostat and SAHA on α-tubulin and histone H4 acetylation levels in K562, KBM5, multiresistant K562-R, and imatinib-resistant KBM5-IR cell lines (Fig. 2C). After 24 h of martinostat treatment, the α-tubulin and histone H4 acetylation levels increased in a dose-dependent manner in all CML cell lines. Interestingly, martinostat (500 nM) displayed acetylation levels comparable to SAHA (5 μM). As α-tubulin is a target of HDAC6 and histone H4 is a target of HDAC1, we performed additional blots confirming that total histone H4 and α-tubulin levels remain unchanged under our treatment conditions (Fig. S3). Our in vitro results validated the in vitro activity assays and confirmed that martinostat inhibits class I and IIb HDACs.

### Martinostat reduces CML cell proliferation and viability

With clear evidence that martinostat increases histone and tubulin acetylation, we evaluated its effect on CML cell proliferation and viability to determine whether these acetylation changes impaired leukemia cell growth. To assess the effects of martinostat, sensitive and resistant K562 and KBM5 cells were treated with increasing concentrations of martinostat for up to 72 h. We used the FDA-approved pan-HDACi SAHA as a reference. Martinostat treatment reduced the proliferation and viability of CML cell types, as shown by trypan blue staining assays. The human non-cancerous cell line RPMI-1788 was treated with martinostat under similar conditions. The viability and proliferation of martinostat-treated RPMI-1788 cells were also affected in a dose-dependent manner. The concentrations that reduced cell viability by 50% (LD_50_) and growth by 50% (GI₅₀) are listed in Table 4. To assess the differential targeting effects of martinostat on CML cells versus healthy cells, we calculated selectivity factors for martinostat and SAHA (Table 5). Martinostat exhibited an increased selectivity factor of 39.0 ± 0.9 for proliferation inhibition in K562-R cells, compared to SAHA’s selectivity factor of 1.0 ± 0.5. This indicates that martinostat effectively targeted the proliferation of resistant leukemia cells, which is a notable advantage over SAHA. Moreover, in terms of cell viability, martinostat achieved a high selectivity factor of 27.7 ± 0.9, compared to SAHA, which displayed a factor of only 0.4 ± 0.3. In the KBM5 cell line, martinostat also demonstrated higher selectivity, achieving a viability selectivity factor of 67.1 ± 1.0 compared to SAHA’s 2.6 ± 0.5 selectivity. This substantial difference highlights the potential of martinostat to selectively reduce leukemia cell viability, while sparing normal cells. Additionally, the selectivity factor for proliferation inhibition in KBM5 cells was 26.7 ± 1.0, exceeding SAHA’s factor of 1.9 ± 0.8. These results demonstrate that martinostat provides a significant selectivity advantage over SAHA in CML models, particularly in drug-resistant K562-R and highly proliferative KBM5 cells. These highly selective factors for cell viability and proliferation suggest that martinostat may offer a more targeted therapeutic approach for CML.

**Table 4:** Effect of martinostat and SAHA on the proliferation and viability of imatinib-sensitive and -resistant CML cells and normal RPMI-1788 cells

Compound	µM	Time	Cell model				
			K562	K562-R	KBM5	KBM5-IR	RPMI-1788
Martinostat	GI_50_^a^	24 h	0.22 ± 0.15	0.04 ± 0.02	0.05 ± 0.03	0.14 ± 0.09	1.42 ± 1.20
		48 h	0.08 ± 0.04	0.05 ± 0.01	0.04 ± 0.02	0.06 ± 0.02	0.20 ± 0.10
		72 h	0.13 ± 0.07	0.07 ± 0.05	0.09 ± 0.03	0.08 ± 0.04	0.17 ± 0.04
	LD_50_^b^	24 h	> 10	> 10	> 10	> 10	> 10
		48 h	3.26 ± 0.47	1.79 ± 0.42	0.74 ± 0.33	5.14 ± 1.72	> 10
		72 h	1.29 ± 0.10	0.70 ± 0.05	0.22 ± 0.03	0.37 ± 0.03	2.54 ± 0.63
SAHA	GI_50_	24 h	0.63 ± 0.54	7.53 ± 0.95	3.82 ± 2.47	3.26 ± 3.09	7.37 ± 3.83
		48 h	0.69 ± 0.44	1.49 ± 0.31	0.79 ± 0.13	1.59 ± 0.31	2.84 ± 0.19
		72 h	0.91 ± 0.24	1.44 ± 0.14	1.09 ± 0.16	1.55 ± 0.11	1.88 ± 0.27
	LD_50_	24 h	> 50	> 50	> 50	> 50	> 50
		48 h	31.96 ± 16.65	42.01 ± 12.40	6.14 ± 2.96	11.38 ± 9.57	16.15 ± 2.75
		72 h	6.62 ± 1.99	7.80 ± 0.84	2.67 ± 0.84	3.03 ± 0.39	4.42 ± 0.07

Cells were treated with a range of concentrations of martinostat or suberoylanilide hydroxamic acid (SAHA), and proliferation and viability were evaluated after 24, 48, and 72 h of incubation

^a^Growth inhibition (GI)_50_: Drug concentration responsible for inhibiting 50% of the growth of a specified cell line (mean ± SD of three independent experiments)

^b^Lethal dose (LD)_50_: Drug concentration responsible for inhibiting the viability of the specified cell line by 50% (mean ± SD of three independent experiments)

**Table 5:** Compared to SAHA, martinostat displays a better selectivity for chronic myeloid leukemia cells versus normal cells

CML model	Selectivity factor			
	Proliferation^a^		Viability^b^	
	Martinostat	SAHA	Martinostat	SAHA
K562	6.4 ± 1.1	11.7 ± 1.0	15.2 ± 0.9	0.5 ± 0.5
K562-R	39.0 ± 0.9	1.0 ± 0.5	27.7 ± 0.9	0.4 ± 0.3
KBM5	26.7 ± 1.0	1.9 ± 0.8	67.1 ± 1.0	2.6 ± 0.5
KBM5-IR	10.0 ± 1.0	2.3 ± 1.1	9.7 ± 1.0	1.4 ± 0.9

^a^Selectivity factor as the ratio growth inhibition (GI)_50_(RPMI1788)/GI_50_[chronic myeloid leukemia (CML)] at 24 h

^b^Selectivity factor as the ratio lethal dose (LD)_50_(RPMI1788)/LD_50_(CML) at 48 h

**Fig. 3:**
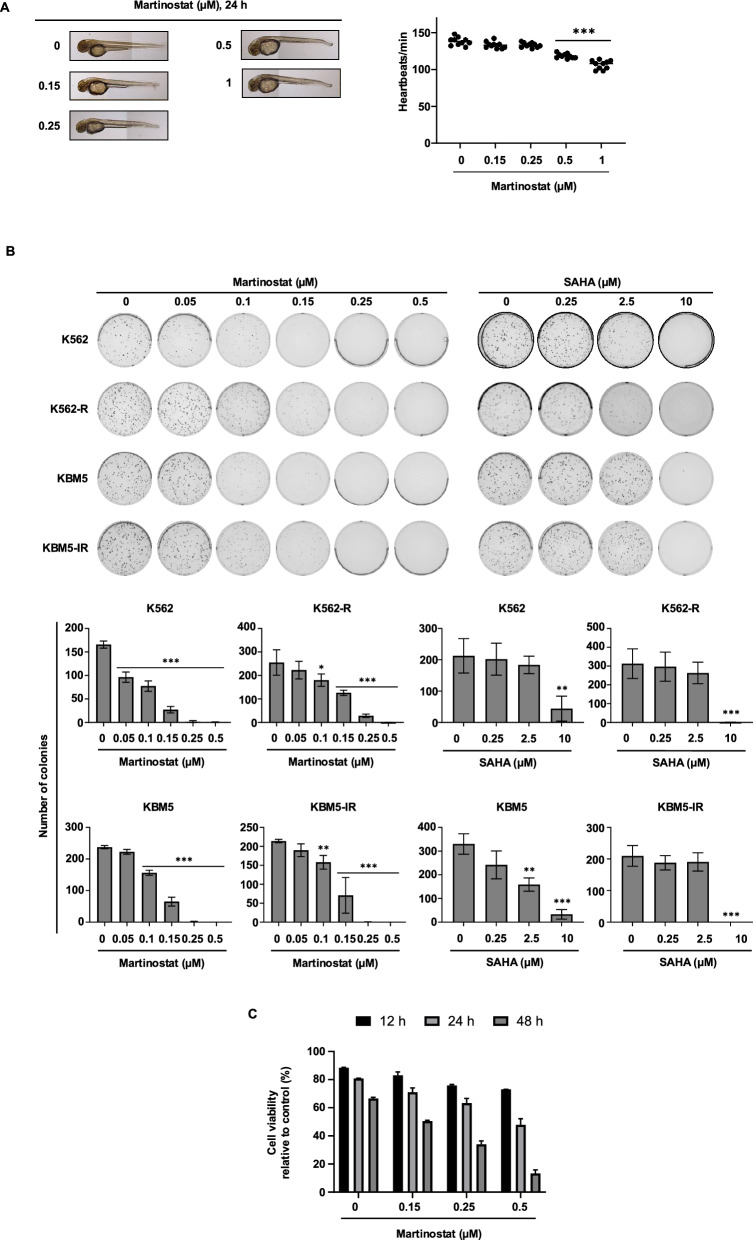
Anti-Leukemic Effect of the HDAC Inhibitor martinostat in Imatinib-Sensitive and -Resistant CML Cells. **A** Zebrafish embryos (*n* = 10 per group) were incubated with the indicated concentrations of martinostat. After 24 h, fish were photographed to document the morphology (left panel), and the heartbeat frequency was monitored (right panel). **B** Colony formation assays with the indicated imatinib-sensitive and -resistant CML cells after treatment with martinostat and SAHA at the indicated concentrations for 10 days. Upper panel: representative images of three independent assays and the corresponding quantifications of the number of colonies (lower panel). **C** Viability graph of PBMCs from two CML patients after treatment with increasing concentrations of martinostat for up to 48 h. Graphs are the mean ± SD. *P* values were determined by one-way ANOVA with Dunnett’s multiple comparison test. *, **, and *** indicate *P* < 0.05, *P* < 0.01, and *P* < 0.001, respectively, versus control cells. A.u.: arbitrary units

To evaluate the effects of martinostat at the earliest stages of embryonic development and provide key insights into potential off-target effects, allowing us to identify safety concerns that may affect drug candidates in the initial phases of development, we conducted zebrafish larval toxicity assays (Fig. 3A). The results indicated that a 24-h martinostat exposure did not affect larval viability (i.e. any of the fish died) or morphology (i.e., constant morphology of the yolk sac and no sign of axial or tail malformations); the heartbeat rate decreased only at the highest doses tested (0.5 and 1 μM) (Fig. 3A). Therefore, we excluded potential teratogenicity and immediate systemic toxicity.

### Martinostat exhibits anti-leukemic properties in imatinib-sensitive and resistant CML cells

Considering the antiproliferative potential of martinostat in both drug-sensitive and drug-resistant CML cells, we tested its anti-leukemic efficacy and safety using 3D colony assays and CML patient cells. 3D colony formation assays revealed that martinostat induced a dose-dependent reduction in colony number by 83.7%, total colony area by 92%, and average colony size by 86.6% at a concentration of 0.15 μM (Fig. 3B, Fig. S4). In ex vivo experiments with primary cells from two patients with CML, martinostat decreased the viability of patient-derived PBMCs in a dose- and time-dependent manner (Fig. 3C).

### Analysis of mRNA sequencing in martinostat-treated cells reveals modulation of cell cycle and apoptosis gene expression profiles

**Fig. 4:**
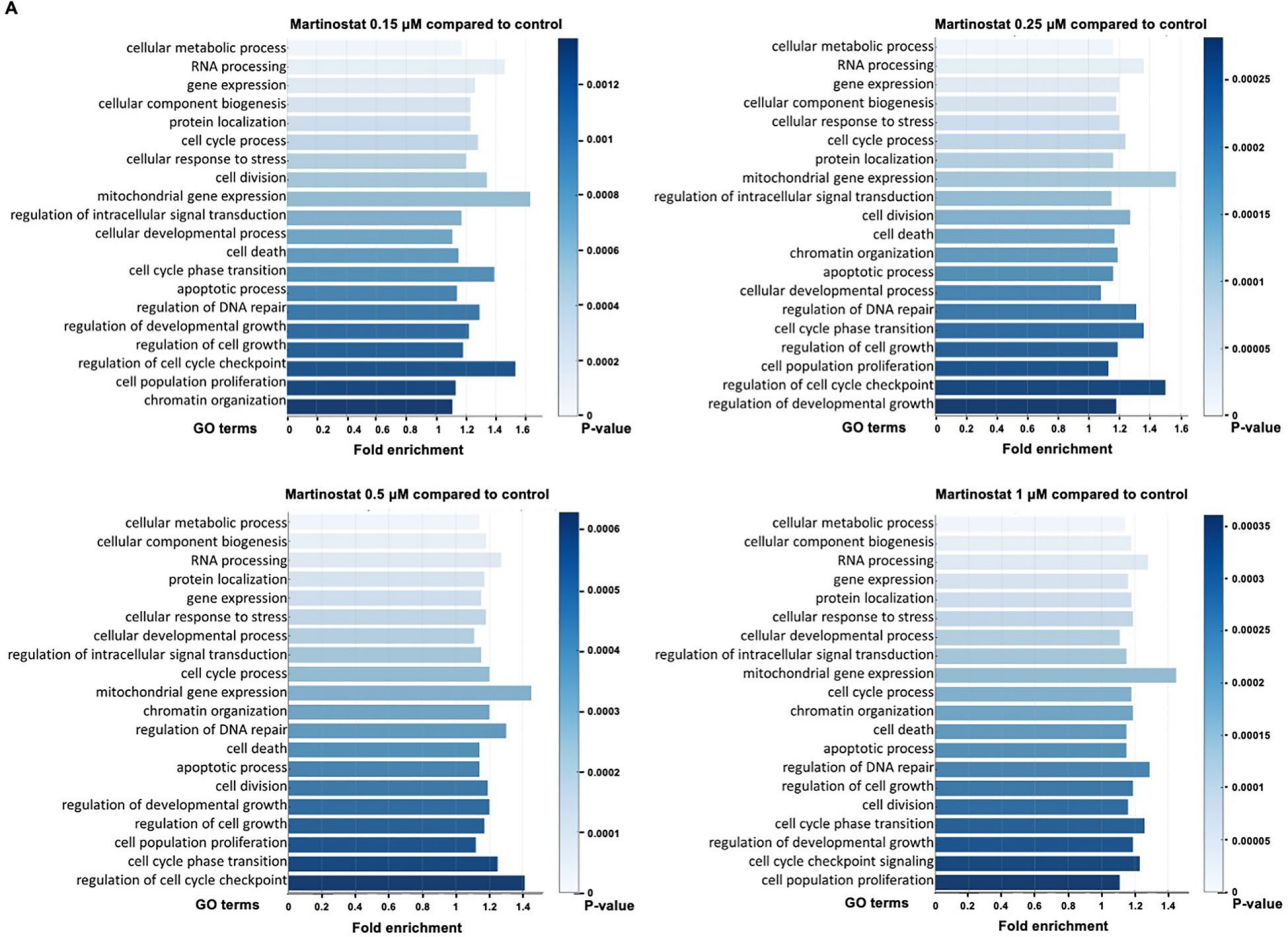

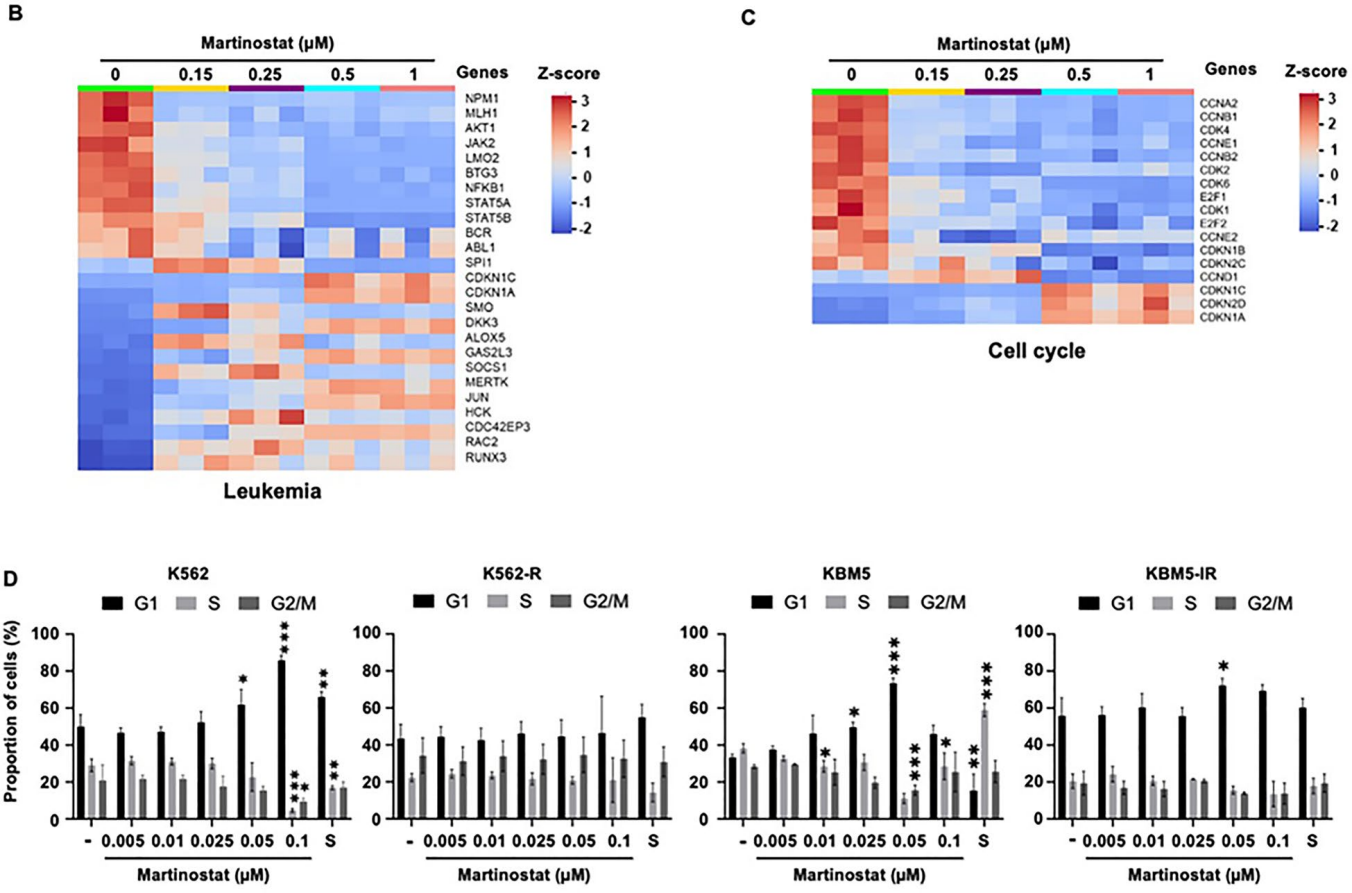
mRNA-sequencing analysis in martinostat-treated K562 cells. **A** Bar graphs of the enrichment scores for gene ontology (GO) terms. GO terms are displayed along the *y*-axis, arranged in descending order of significance, with fold enrichment scores on the *x*-axis. The color gradient represents the statistical significance (*P* value) of each GO term, with darker shades indicating higher significance. **B**, **C** Heatmaps of gene expression patterns for leukemia (**B**) and cell cycle (**C**) categories. The heatmaps illustrate the mRNA levels differentially expressed for the indicated genes as a function of martinostat concentrations. **D** Cell cycle distribution analyses in the indicated CML cell lines treated for 24 h with increasing concentration of martinostat or 2 µM SAHA (S). Data represent the mean ± SD of three independent experiments. *, **, and *** indicate *P* < 0.05, *P* < 0.01, and *P* < 0.001, respectively, versus control cells

To elucidate the molecular pathways underlying the anti-leukemic action of martinostat, we performed transcriptomic analyses to identify changes in cell cycle, apoptosis, and other critical regulatory genes following martinostat treatment. We performed mRNA sequencing (mRNA-seq) after treating K562 cells with 0.15–1 μM martinostat for 24 h. Compared with the control (0 µM martinostat) group, martinostat treatment influenced gene expression in a dose-dependent manner, as demonstrated by the upregulation and downregulation of genes in the volcano plots (Fig. S5A). To investigate the gene ontologies regulated by martinostat treatment, we conducted GO term analysis based on P values lower than 0.05 and a log2 fold enrichment score over 1. The results showed that martinostat treatment regulated the expression of GO categories, including cell cycle, mitochondrial gene expression, apoptosis, cell population, proliferation, and regulation of cell cycle checkpoints (Fig. 4A). Martinostat treatment also differentially regulated the expression of genes belonging to leukemia and cell cycle categories (Fig. 4B, C), and epigenetic and chromatin modification enzymes, DNA repair, and apoptotic processes (Fig. S5B). As cell cycle categories were reported among the top categories regulated by martinostat treatment in GO terms and heatmap graphs, we investigated the mRNA expression levels of cell cycle-regulated genes individually. The results showed a reduced mRNA expression of cyclin-dependent kinases (CDK1, CDK2, CDK4, and CDK6) and cyclins (CCNA2, CCNB1, CCNB2, CCND1, CCNE1, and CCNE2). Conversely, an increased expression of cyclin-dependent kinase inhibitors (CDKN1A, CDKN1B, CDKN1C, CDKN2C, and CDKN2D) was observed (Fig. S6). Considering the modulation of cell cycle regulators at the mRNA level and the fact that martinostat treatment inhibited CML cell proliferation (Table 4), we investigated its impact on cell cycle distribution (Fig. 4D). After 24 h, the G1 phase increased by 1.9-fold in imatinib-sensitive K562 cells. In resistant K562 cells, martinostat did not alter the cell cycle phase distribution. In imatinib-sensitive and imatinib-resistant KBM5 cells, the G1 phase increased 2.1-fold and 1.3-fold, respectively.

**Fig. 5:**
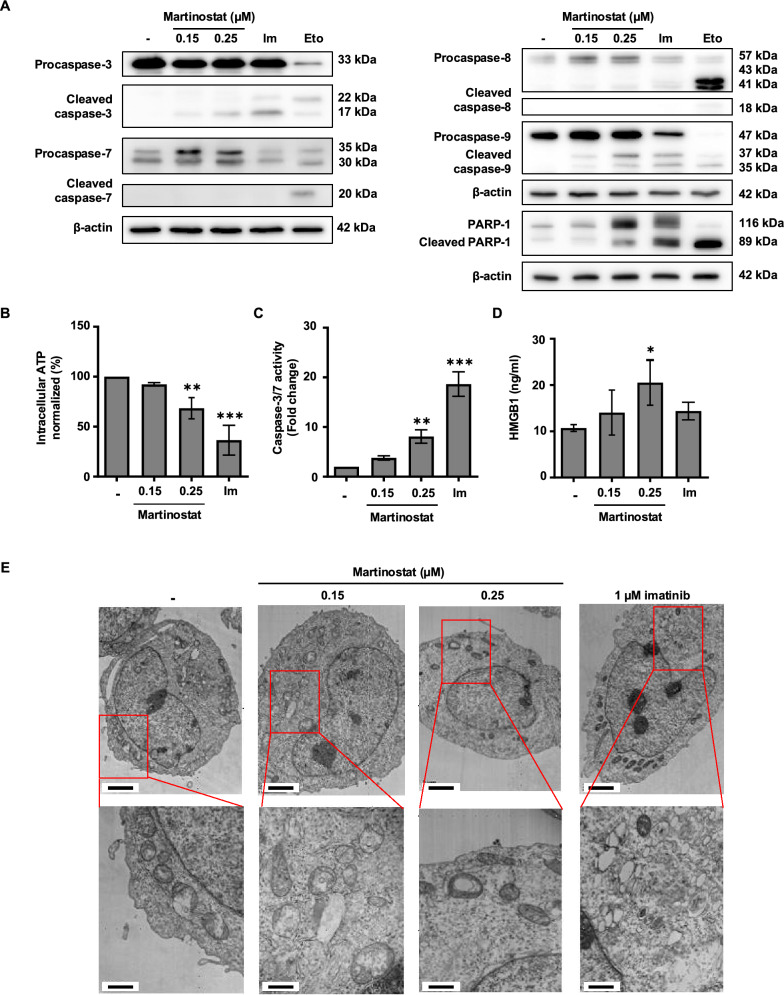
Effect of martinostat on imatinib-sensitive K562 Cells. K562 cells were treated for 24 h with the indicated concentrations of martinostat or 1 µM imatinib (Im). **A** Analysis of caspases and poly(ADP-ribosyl)transferase (PARP)−1 cleavage by western blot analysis after 24 h of treatment. U937 cells treated with 40 μM etoposide (Eto) for 4 h were used as a positive control for caspase and PARP-1 cleavage. β-actin was used as a loading control in western blot analysis. Immunoblot pictures are representative of three independent experiments. Quantification of **B** intracellular ATP levels, **C** caspase-3/7 activity, and **D** high mobility group box 1 (HMGB1) release. Data are the mean ± SD of three independent experiments. *P* values were calculated by one-way ANOVA with Dunnett’s multiple comparisons test. *, ** and *** indicate *P* < 0.05, *P* < 0.01 and *P* < 0.001, respectively, versus control cells. **E** Representative images of electron microscopy pictures. The scale bar of the upper images indicates 2 μm, and the scale bar of the lower images indicates 0.5 μm

Considering that mRNA-seq results showed that the essential factors of the cell death category were modulated by martinostat (Fig. S5B), we investigated the activation of apoptotic effectors in the CML cells. Western blot analysis confirmed that martinostat treatment increased the cleavage of caspase-3, caspase-9, and poly (ADP-ribose) polymerase-1 (PARP-1) in K562 cells (Fig. 5A). Consistent with these results, 0.15 and 0.25 μM martinostat decreased intracellular ATP levels by 7.6% and 31.6%, respectively (Fig. 5B), and increased caspase-3/7 activity by 1.9-fold and 4.0-fold (Fig. 5C). We also assessed the release of HMGB1, which increased by 1.5- and 3.3-fold at 0.15 and 0.25 μM, respectively, in K562 cells (Fig. 5D). HMGB1 release increased by 1.5-fold in imatinib-resistant KBM5 cells (Fig. S7).

Furthermore, mRNA-seq data revealed the modulation of the autophagic stress response. At low concentrations, we observed that partial HDAC inhibition disrupted the transcriptional complexes required for the proper expression of autophagy- and apoptosis-related genes, leading to the downregulation of key regulators involved in autophagosome formation, membrane dynamics, and cell death control, likely due to impaired transcription factor recruitment, altered cofactor binding, and unstable regulatory complexes. At higher concentrations, autophagy and lysosome biogenesis genes, such as SQSTM1/p62, LAMP2, and PINK1, were robustly upregulated along with tumor suppressors, such as PTEN, and pro-apoptotic factors, including BAD and BIK, which reinforce autophagic flux and promote cellular homeostasis (Fig. S8). We confirmed these observations using transmission electron microscopy to investigate the effect of martinostat on the morphology of imatinib-sensitive K562 cells after 24 h of treatment with martinostat (Fig. 5E). Martinostat treatment results in abnormal cell morphology and increased autophagosome formation. Similar morphological changes were observed in the other imatinib-sensitive and imatinib-resistant CML cell lines (Fig. S9), consistent with the onset of apoptotic cell death.

### Induction of cell death by martinostat and imatinib in CML Cells

**Fig. 6:**
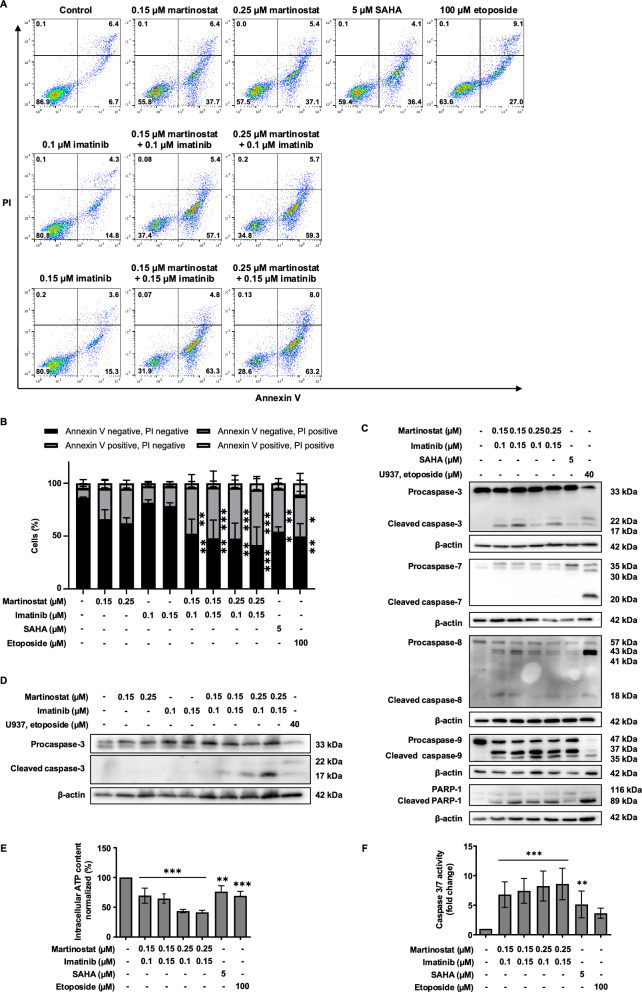
Martinostat combined with imatinib induces a synergistic effect in imatinib-sensitive K562 cells. K562 cells were treated with the indicated compounds for 24 h. **A** Flow cytometry analysis after annexin V and propidium iodide (PI) staining is shown as representative dot plots of three independent experiments, including the percentage of events in each quadrant and corresponding quantification (**B**). **C** Analysis of caspase and poly(ADP-ribosyl)transferase (PARP)−1 cleavage using western blotting. **D** Analysis of caspase-3 cleavage by martinostat, imatinib, or their co-treatment using western blotting. U937 cells treated with 40 μM etoposide for 4 h were used as positive controls for caspase and PARP-1 cleavage. β-actin was used as a loading control in western blot analysis. Immunoblotting images are representative of three independent experiments. Quantification of **(E)** intracellular ATP levels and **F** caspase-3/7 activity. Data are presented as the mean ± SD of three independent experiments. *P* values were calculated using one-way analysis of variance (ANOVA) with Dunnett’s multiple comparison test. *, ** and *** indicate *P* < 0.05, *P* < 0.01 and *P* < 0.001, respectively, versus control cells

As martinostat modulates the expression of key apoptosis and autophagy regulators, we next explored whether its co-administration with imatinib amplifies cell death signals in both sensitive and resistant CML cell lines. First, we investigated the effect of martinostat and imatinib co-treatment on K562 cell death. Flow cytometry analysis showed that co-treatment induced more annexin V-positive cells than treatment with either drug alone (Fig. 6A, B). Based on these results, we calculated the combination index (CI), which indicated synergism between the four combinations of martinostat and imatinib (Table S3).

Co-treatment also induced cleavage of pro-caspase-3, −8, −9, and PARP-1, indicating caspase-dependent apoptosis (Fig. 6C, D). Additionally, intracellular ATP content decreased by 30.5% (Fig. 6E) with the co-treatment of 0.15 μM martinostat and 0.1 μM imatinib, while caspase-3/7 activity increased 6.8-fold under the same conditions (Fig. 6F).

**Fig. 7:**
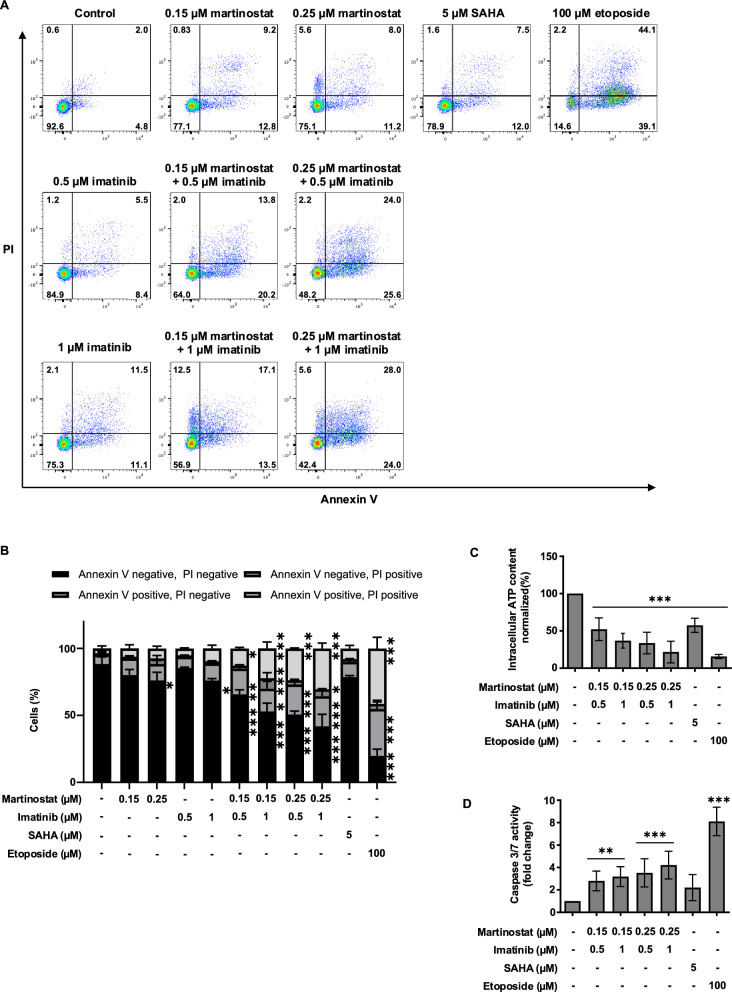
Martinostat combined with imatinib induces a synergistic effect in resistant K562 cells. K562-R cells were treated with the indicated compounds for 96 h. **A** Flow cytometry analysis after annexin V and propidium iodide (PI) staining is shown as representative dot plots of three independent experiments, including the percentage of events in each quadrant and corresponding quantification (**B**). Quantification of annexin V and PI staining. Quantification of intracellular ATP levels (**C**) and caspase-3/7 activity (**D**). Data are presented as the mean ± SD of three independent experiments. *P* values were calculated using one-way analysis of variance (ANOVA) with Dunnett’s multiple comparison test. *, ** and *** indicate *P* < 0.05, *P* < 0.01 and *P* < 0.001, respectively, versus control cells

Next, we extended our findings by testing a combination of resistant K562 (K562-R) cell lines. Similar to that observed in the sensitive cell line, this combination induced more annexin V-positive cells than either drug alone (Fig. 7A, B). Based on these results, we calculated the combination index that revealed synergism using four combinations of martinostat and imatinib in the K562-R cells (Table S3). Furthermore, co-treatment of 0.25 µM martinostat imatinib with 1 µM) decreased intracellular ATP by 82% (Fig. 7C) and increased caspase-3/7 activity by 4.2-fold (Fig. 7D) compared to the control.

**Fig. 8:**
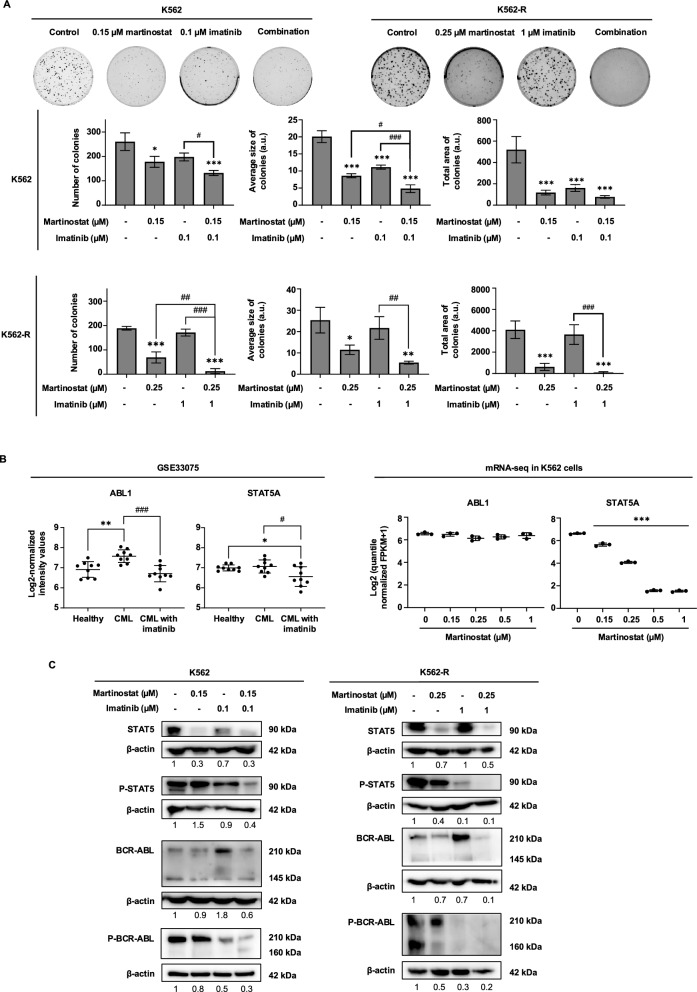
Martinostat combined with imatinib inhibits colony-forming capacity and the BCR-ABL signaling pathway in imatinib-sensitive and -resistant K562 cells. **A** Colony formation assays with the indicated imatinib-sensitive and -resistant CML cells after treatment with martinostat alone or combined with imatinib at the indicated concentrations for 10 days. Upper panels: representative images of three independent assays and the corresponding quantifications (lower panels) of the number of colonies, average size of colonies, and total area of colonies scored after MTT addition. Data are the mean ± SD of three independent experiments. **B** ABL proto-oncogene 1, non-receptor tyrosine kinase (ABL1), and signal transducer and activator of transcription 5 (STAT5A) mRNA expression levels in the GSE33075 dataset and mRNA-sequencing data from K562 cells treated for 24 h with increasing concentrations of martinostat. For mRNA-sequencing data, gene expression levels were compared based on normalized fragments per kilobase of transcript per million mapped reads (FPKM). Data are the mean ± SD. **C** Western blot analysis of STAT5 and P-STAT5 as well as BCR-ABL and P-BCR-ABL in K562 and K562-R cells treated for 24 and 96 h, respectively, with martinostat alone or combined with imatinib. β-actin was used as a loading control. Immunoblot pictures are representative of three independent experiments, and their quantifications are indicated under the blots. *P* values were measured by one-way ANOVA with Šídák’s multiple comparisons test. *, ** and *** indicate *P* < 0.05, *P* < 0.01 and *P* < 0.001, respectively, versus control cells. #, ## and ### indicate *P* < 0.05, *P* < 0.01 and *P* < 0.001 for the indicated comparisons; a.u.: arbitrary units

Finally, we confirmed the increased anti-CML potential of the combination of martinostat and imatinib by using 3D colony formation assays (Fig. 8A). In K562 cells, co-treatment significantly decreased the number of colonies, total colony area, and average colony size by 49.2%, 85.1%, and 75.9%, respectively, compared with the control group. In K562-R cells, the combination treatment significantly reduced the number of colonies, total colony area, and average colony size by 93.2%, 98%, and 70.4%, respectively. Similar findings were observed in KBM5 and MEG01 CML cells, demonstrating significant inhibition of colony metrics (Fig. S10).

To better characterize the K562-R cell line, we performed differential expression and enrichment analyses of differentially expressed genes. We identified 1681 upregulated and 837 downregulated genes in K562-R cells compared to K562 cells (P value < 0.05, |log_2_FC|> 2, Tables S4, S5). The differentially expressed genes are presented in a Volcano plot and a heatmap in Fig. S11A, B. The top Gene Ontology (GO) terms associated with upregulated and downregulated genes (based on over-representation analysis) are shown in Supplementary Fig. S12. GO-enriched pathways are summarized in Tables S6, S7, respectively. We additionally conducted KEGG pathway enrichment analysis, which is provided in Table S7 alongside GO pathway analysis results.

### Martinostat and imatinib co-treatments inhibit the BCR-ABL signaling pathway in imatinib-sensitive and -resistant K562 Cells

As martinostat and imatinib co-treatment synergistically induced apoptosis in CML cells, we investigated how this combination influenced the BCR-ABL signaling pathway, a principal driver of CML pathogenesis. To assess the effects of TKIs and HDACi on BCR-ABL-related genes, we compared the expression of ABL1 and STAT5A between two datasets: the gene expression dataset GSE33075 and mRNA-sequencing data from CML K562 cells treated with martinostat (Fig. 8B). In GSE33075, CML patients treated with imatinib showed significantly lower expression of ABL1 and STAT5A than the healthy group and the untreated CML group. In martinostat-treated K562 cells, mRNA sequencing revealed a significant concentration-dependent decrease in STAT5A gene levels. The downregulation of proliferative and survival signaling components at low concentrations, including JAK2, STAT5A/B, BCR, ABL1, and PIK3CA, may be complemented by imatinib, thereby preventing or limiting the ability of cells to rely on growth signals (Fig. S13). In imatinib-sensitive K562 cells, co-treatment reduced the expression of STAT5 and its phosphorylation by 70.2% and 64.4%, respectively, without changing BCR-ABL expression levels (Fig. 8C). In resistant K562 cells, the expression levels of BCR-ABL and phosphorylated BCR-ABL were decreased by 96% and 84%, respectively, whereas STAT5 and its phosphorylation were reduced by 54.5% and 87.9%, respectively (Fig. 8C).

### In vivo anticancer effects of martinostat and imatinib co-treatment

**Fig. 9:**
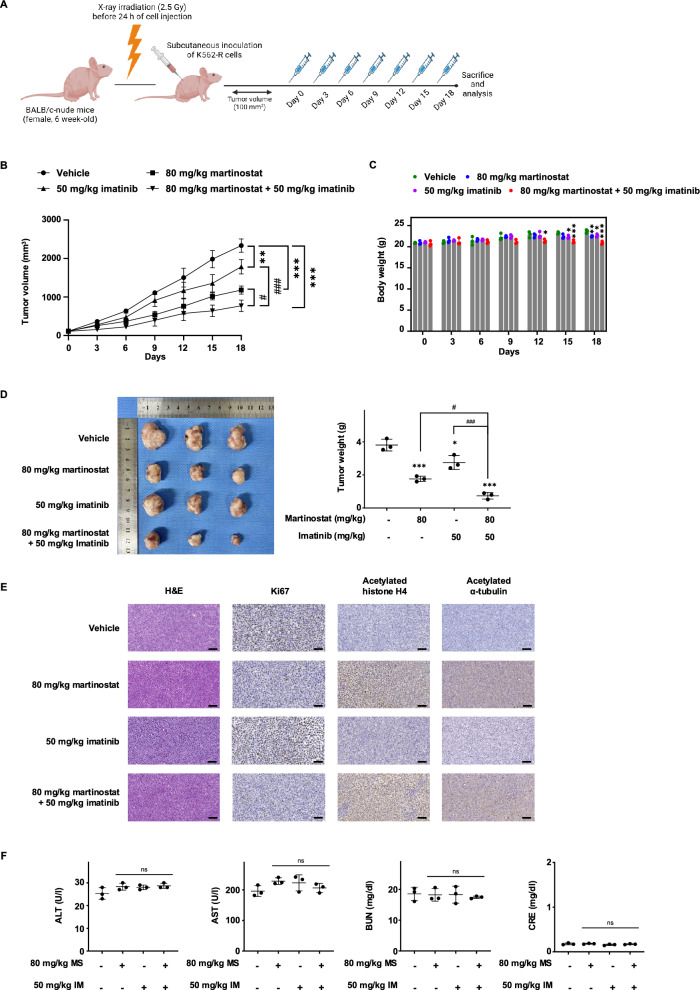
Martinostat Combined with Imatinib Enhances Anticancer Activity in K562-R–Bearing BALB/c Nude Mice. **A** Experimental scheme of K562-R cell xenografts in irradiated BALB/c nude mice (*n* = 3 per group). Martinostat (MS) (80 mg/kg) and Imatinib (IM) (50 mg/kg) were injected intraperitoneally in corn oil/10% DMSO every three days. **B** Tumor volume was assessed by caliper measurement across the 18 days of the experiment. **C** Body weight was measured throughout the 18-day experiment. **D** Photographs (left panel) of tumors excised from K562-R xenograft mice and their corresponding weights (right panel) after sacrifice. **E** After 18 days, animals were sacrificed, and hematoxylin and eosin (H&E) staining and immunohistochemistry (IHC) staining of tumor sections with anti-Ki67, anti-acetylated histone H4, and anti-acetylated α-tubulin were performed. Pictures (magnification: × 20) are representative of the observations reported in all animals. The scale bar indicates 50 μm. **F** Results of biochemical blood analyses for alanine aminotransferase (ALT), aspartate aminotransferase (AST), blood urea nitrogen (BUN), and creatinine (CRE) levels. All graphs represent the mean ± SD. *P* values were determined by one-way ANOVA with Dunnett's (**B**, **C**) or Šídák's (**D**, **F**) multiple comparison tests. *, **, ***, and ns indicate *P* < 0.05, *P* < 0.01, *P* < 0.001, and not significant, respectively, versus vehicle control. #, ## and ### indicate *P* < 0.05, *P* < 0.01 and *P* < 0.001 for the indicated comparisons

Having demonstrated that the combination of martinostat and imatinib disrupted BCR-ABL signaling and significantly reduced cell viability in vitro, we used an in vivo xenograft model to confirm the therapeutic potential and safety profile of this combination. X-ray-irradiated BALB/c nude mice were inoculated with resistant K562 cells, and treatments were administered at the indicated dosages (Fig. 9A). Co-treatment inhibited tumor growth and reduced tumor volume by 67%, 34.6%, and 56.8%, respectively, compared with the vehicle, martinostat, and imatinib groups (Fig. 9B, Fig. S14). Tumor weight was reduced by 80.7%, 58.2%, and 73.3% compared with that in the respective groups (Fig. 9D), confirming the anticancer properties of the combination therapy.

Over the treatment period, the combination therapy group showed a moderate reduction in body weight gain compared to the vehicle group (Fig. 9C). Histological evaluation of tumor tissues via immunohistochemistry indicated that co-treatment significantly increased histone H4 and α-tubulin acetylation in tumors. The proliferation marker Ki67 was significantly decreased, indicating an antiproliferative effect of martinostat alone or combined with imatinib (Fig. 9E, Fig. S15).

Biochemical blood analysis revealed that the treatments did not alter ALT, AST, BUN, or CRE levels (Fig. 9F). This indicated no evidence of organ injury induced by martinostat, imatinib, or their combination, underscoring the safety of combining martinostat and imatinib for further investigations.

## Discussion

The role of HDACs in CML pathogenesis has been established [7]. Our analysis of the GSE5550 patient dataset confirmed significant upregulation of HDACs 1, 2, 3, 6, and 7 in CML LSCs compared with healthy controls. Similarly, HDACs 1, 2, and 3 were upregulated in the GSE97562 dataset [10]. Notably, imatinib-treated patients showed reduced mRNA levels of HDACs 2, 6, and 8, linking HDAC overexpression to BCR-ABL-driven CML proliferation in vivo and supporting a dual-target strategy for HDACs and BCR-ABL.

In vitro, martinostat demonstrated superior efficacy than SAHA in inhibiting HDAC classes I, IIb, and IV. Docking studies confirmed the pan-HDAC inhibitory effect with low binding affinity energies for HDACs 1, 2, 4, 6, 7, and 10. Martinostat exhibited lower IC₅₀ values for specific HDACs and total HDAC inhibition than SAHA, consistent with prior findings of class I and HDAC6 inhibition [15]. Our results further confirmed the inhibition of HDAC classes II and IV by martinostat, using docking and in vitro assays, establishing martinostat as a potent pan-HDACi.

Martinostat increased histone and non-histone acetylation more effectively than SAHA in both imatinib-sensitive and imatinib-resistant CML cells and was active at lower concentrations, potentially reducing SAHA-associated side effects and highlighting the potential of martinostat as an HDACi. Additionally, martinostat met drug-likeness criteria, similar to trichostatin A (TSA) and valproic acid (VPA) [33], further supporting its suitability for therapeutic investigations.

Previously, Wang et al. measured the activity of ^11^C-martinostat against HDACs 1–9 compared to that of SAHA [15], whereas Strebl et al. assessed martinostat against HDAC1, 2, 6, and 8 without using SAHA as a reference [34]. We expanded on prior work by comparing the inhibitory effect of martinostat on the activity of HDAC1 through HDAC11 with that of SAHA under identical conditions. Moreover, we extended our previous docking study by comparing the binding efficacy of martinostat to HDAC2 with that of other HDACi [35]. Our comparative analysis showed that martinostat has a higher binding affinity than SAHA for HDAC1, HDAC2, HDAC3, HDAC6, and HDAC10, which matches our comparative HDAC inhibition results. Additionally, the effect of martinostat on protein acetylation has not yet been tested in CML cells. Our study also represents the first kinetic analysis to describe acetylation stability and cellular responses, addressing the transient acetylation effects seen with some HDACi [36]. In addition, investigating the effect of a given HDACi in various cell models, including hematological models, helps understand cellular responses, particularly in resistant cell models.

Martinostat reduced the proliferation and viability of imatinib-sensitive and -resistant BCR-ABL-positive CML cells in a dose-dependent manner. Differential toxicity tests using non-cancerous RPMI-1788 cells demonstrated the strong selectivity of martinostat for CML cells, which exceeded that of SAHA. Furthermore, martinostat did not affect the viability of the zebrafish larvae. Acute effects on early zebrafish development were assessed to evaluate potential teratogenicity and systemic toxicity. Given the high sensitivity of zebrafish embryos to toxic agents and their genetic similarity to humans (80%) [37], these findings support the stability and safety profile of martinostat. Our results suggest that martinostat is a non-toxic strategy for overcoming imatinib resistance in CML patients.

Imatinib resistance often results from BCR-ABL1 kinase domain mutations that reduce drug binding but can also arise through alternative mechanisms, such as efflux pump overexpression, activation of bypass signaling pathways (e.g., JAK/STAT, PI3K/AKT, RAS/RAF/MEK/ERK), or increased anti-apoptotic proteins (e.g., BCL2 family). Resistant cells may adopt stem cell-like phenotypes or transcriptional states that enhance their survival and drug tolerance. Unlike targeted TKIs, martinostat, a pan-HDACi, does not directly block BCR-ABL1 kinase activity or bypass the pathways. Instead, as shown by our mRNA-seq data, it alters the epigenetic landscape by promoting histone and non-histone protein acetylation, broadly affecting gene transcription. By increasing histone acetylation, martinostat can restore pro-apoptotic factors (e.g., BAD and BIK) and tumor suppressors (e.g., PTEN), suppress pro-survival genes, and restore critical pathways. This reprogramming impairs the ability of cells to sustain alternative growth and survival mechanisms, enhancing their susceptibility to apoptosis in combination with imatinib or other TKIs.

SAHA has been reported to reduce tumor metastasis and volume in breast cancer at 25 mg/kg without altering liver and kidney function indicators (ALT, AST, BUN, CRE) [38]. Even at 50 mg/kg, SAHA showed no toxicity in non-small-cell lung cancer models [39]. Similarly, in our in vivo assay, 80 mg/kg martinostat did not affect these biochemical parameters, and its combination with imatinib showed no signs of organ toxicity, confirming the safety of martinostat alone or in combination with imatinib.

In a clinical trial, oral SAHA demonstrated anti-leukemic activity in advanced leukemia and MDS, with histone H3 acetylation observed in the blood and BM blasts [40]. The MTD for oral SAHA was 200 mg twice daily or 250 mg three times daily for 14 days in a 21-day cycle, with side effects such as gastrointestinal issues and fatigue resolving within 2 weeks. Although SAHA is non-toxic in mouse models at 25–50 mg/kg, martinostat showed similar safety at 80 mg/kg, suggesting that its side effects may be milder in future clinical trials. This finding supports further investigations of martinostat and imatinib as combination therapies to overcome TKI resistance in CML.

Additionally, martinostat inhibited colony formation and cell cycle progression more effectively than SAHA did, inducing G1 arrest in imatinib-sensitive and imatinib-resistant CML cells. It reduces the mRNA levels of cell cycle–promoting cyclins (CCNA2, CCNB1, CCNB2, CCND1, CCNE1, and CCNE2) and upregulates the levels of cyclin-dependent kinase inhibitors (CDKN1A, CDKN2C, and CDKN2D). Similar effects have been reported for SAHA, which downregulates cyclin D1 and upregulates CDKN1A and CDKN1B in rhabdomyosarcoma models [41], indicating that HDACi broadly regulate cell cycle-associated genes. HDAC classes I and II, such as CDKN2A, CDKN1A, and CDKN1B, contribute to transcriptional repression of CDK inhibitor genes. HDACs 3 and 4 repress p21 expression in colon cancer [42–44]. In hepatocellular carcinoma, inhibition of HDACs 1 and 2 by SAHA increases p19 and p21 expression, inducing apoptosis [45]. Similarly, FDA-approved HDACi (panobinostat, SAHA, belinostat, VPA) repressed HDACs 1 and 2, with panobinostat-mediated p21 upregulation leading to cell cycle inhibition and cell death in K562-R cells [46]. These findings suggest that martinostat effectively targets the HDACs involved in cell cycle progression and proliferation in imatinib-sensitive and -resistant CML cells. The effects of martinostat on K562-R cells involve epigenetic reprogramming, rather than classical cell cycle checkpoint arrest. Instead of phase-specific accumulation (e.g., G1 or S phase), martinostat slows cell proliferation by altering gene expression, reducing critical growth factors, impairing metabolism, and disrupting DNA repair. These changes reduced the cell viability and proliferation, as reflected by the lower GI_50_ values.

Martinostat‐mediated modulation of STAT5 operates on two mechanistically distinct tiers. First, we observed a net decline in total STAT5 abundance. HDAC inhibitors such as martinostat are known to increase the acetylation of non-histone substrates, marking them for proteasomal turnover; the resulting loss of STAT5 protein has been reported for several pan-HDAC inhibitors in leukemic settings [8]. Second, the residual STAT5 molecules display enhanced phosphorylation on the canonical activation tyrosine. This apparent paradox is readily explained by compensatory signaling: HDAC blockade can relieve negative-feedback loops (e.g., SOCS upregulation or SHP-1/2 recruitment) and promote JAK2 activity, thereby elevating the specific activity of the remaining STAT5 pool. Consequently, martinostat imposes selective stress that simultaneously depletes STAT5 protein and activates the fraction that escapes degradation, yielding the opposite changes in the two readouts.

Martinostat treatment of K562 cells reduced STAT5, a key downstream target of BCR-ABL that is critical for leukemogenesis. STAT5 and its target ID-1 require HDAC recruitment for transcriptional activation [47, 48]. Consistent with this, the HDACi trichostatin A (TSA) inhibited STAT5-mediated transcription in Ba/F3 cells [49], whereas SAHA, alone or in combination with the JAK2 inhibitor ruxolitinib, reduced phosphorylated STAT5 in Philadelphia chromosome-negative neoplasms [50]. In K562 cells, SAHA suppresses STAT5 expression and phosphorylation [51]. Treatment with the HDACi MAKV-8, alone or combined with imatinib, suppressed STAT5 expression and phosphorylation in K562 cells [10]. Similarly, co-treatment with martinostat and imatinib further reduced BCR-ABL and STAT5 expression in both imatinib-sensitive and imatinib-resistant K562 cells, suggesting that martinostat is a potential therapeutic candidate for CML, either alone or with TKIs. Interestingly, martinostat increased the phosphorylation of STAT5 and BCR-ABL despite reducing the total protein levels, possibly reflecting a compensatory mechanism. This may be because of the dynamic interplay between STAT5 acetylation and phosphorylation. As an HDACi, martinostat enhances the acetylation of histones and non-histone proteins, including STAT5, which can increase DNA-binding and transcriptional activity. Phosphorylated and acetylated STAT5 dimerizes, translocates to the nucleus, and activates transcription [52]. Acetylation may also facilitate interactions with upstream kinases, thereby promoting STAT5 phosphorylation. For BCR-ABL, acetylation of regulatory proteins in the signaling pathway can stabilize its active phosphorylated form, thereby maintaining survival signaling under therapeutic stress. Combination therapy with HDACi and TKIs has demonstrated synergistic effects in CML: SAHA, combined with dasatinib, depleted BCR-ABL, and STAT5 in K562, LAMA-84, and BaF3 cells [14]. Other studies reported synergy between novel HDACi and imatinib [10, 12, 53], while panobinostat combined with ponatinib showed efficacy against imatinib-sensitive and -resistant CML by targeting BCR-ABL and STAT5 [54].

Similarly, martinostat demonstrated synergy with imatinib by inducing caspase-dependent apoptosis. As imatinib induces apoptosis via caspase activation [55], martinostat enhances this effect, supporting HDACi and TKI combination therapy to overcome TKI resistance and achieve CML cytotoxicity.

Our mRNA-seq data revealed that varying intensities of HDAC inhibition induced cellular state shifts, providing a rationale for incorporating autophagy inhibitors. Low-level HDAC inhibition disrupts pro-survival and autophagy-related genes, sensitizing cells to apoptotic stimuli. In this early stage, autophagy inhibitors prevent stress-adaptive autophagy and enhance cell death. At higher HDACi doses, the cells mount a robust survival response via TFEB-driven autophagy and upregulation of lysosomal and pro-apoptotic regulators. Blocking this compensatory autophagic pathway at this stage shifts the balance toward apoptosis. Combining HDAC inhibition with autophagy blockade or pro-apoptotic interventions, such as imatinib, maximizes cytotoxicity by counteracting adaptive survival mechanisms. These findings align with those of Xin et al., who reported that treatment with a PI3K/AKT/mTOR inhibitor combined with imatinib-enhanced autophagy, while dysregulating CML proliferation [56]. This finding supports the potential of autophagy inhibitors to enhance martinostat-TKI combination treatment, an area of ongoing research.

Zebrafish models offer rapid in vivo analysis because of their transparency, ease of genetic manipulation, and cost-effectiveness. However, their physiology and drug metabolism differ from that of humans, limiting their direct clinical translation [57]. To address this, we validated our findings using a mouse xenograft model that provides a mammalian physiological environment and pharmacokinetics similar to human patients. In K562-R cell-inoculated BALB/c nude mice, the combination of martinostat and imatinib produced a significantly greater synergistic effect than either compound alone. This aligns with previous findings, such as the synergistic interaction of the HDACi CAY10683 and imatinib in imatinib-resistant K562 cells [53], further supporting the combination of HDACi and TKIs in overcoming resistance in CML.

The broad epigenetic modulation of martinostat has therapeutic potential beyond BCR–ABL–driven leukemia, such as that involving ETV6-ABL1 [58] or NUP214-ABL1 [59] fusion proteins. These malignancies rely on aberrant tyrosine kinase signaling and transcriptional networks to evade growth and apoptosis. By altering histone and transcription factor acetylation, martinostat may downregulate pro-survival genes and enhance TKI sensitivity. In TKI-resistant cases, the ability of martinostat to disrupt compensatory pathways, including alternative signaling routes, anti-apoptotic BCL2 family upregulation, and lineage plasticity, could improve therapeutic outcomes and delay relapse. These observations support further investigations of martinostat in combination with ABL-targeted therapies in other ABL-dependent malignancies.

In addition to the primary goal of this work, we conducted a differential gene expression analysis comparing K562-R and K562 cells. This supplementary analysis provides valuable context by revealing transcriptional changes associated with acquired resistance, highlighting biological processes that may contribute to the drug-resistant phenotype in CML. The updated expression profile depicts a form of CML that evades BCR-ABL inhibition by linking LSC persistence to a remodeled microenvironment. Notably, the upregulation of RAB27B has been associated with enhanced imatinib resistance and may play a role in disease progression [11]. Similarly, NOTCH2 reinforces the Notch/Wnt signaling axis – previously implicated in LSC self-renewal – thereby promoting resistance [12]. Overexpression of MCAM (CD146), commonly found in tumors and endothelial cells, was also observed in the drug-resistant CML model [13]. While not directly driving proliferation, its elevated expression may reflect adaptation to imatinib-induced stress.

These findings contribute to the growing understanding of resistance mechanisms in CML and lay the groundwork for future studies to identify novel therapeutic targets. However, further experimental validation and mechanistic investigation will be essential to confirm the functional significance of these transcriptional changes and translate them into clinical insights.

In conclusion, martinostat functions as a potent pan-HDACi with significant anti-leukemic effects in imatinib-sensitive and resistant CML cells. It modulates BCR-ABL and STAT5 expression, induces apoptosis, and shows synergistic efficacy with imatinib in vitro and in vivo. These results highlight martinostat as a promising candidate for overcoming TKI resistance in CML therapy and justify further preclinical and clinical studies.

## Supplementary Information

Additional file 1.

Additional file 2.

Additional file 3.

## Data Availability

mRNA-sequencing data of martinostat-treated cells are available at the Gene Expression Omnibus (GEO) (GSE292088). mRNA-sequencing data of K562 and K562R cells are available at the Gene Expression Omnibus (GEO) (GSE298575).
