# Supplementary material for: Martinostat as a novel HDAC inhibitor to overcome tyrosine kinase inhibitor resistance in chronic myeloid leukemia
Source: Clin Epigenetics. 2025 Jul 16;17:125. doi: 10.1186/s13148-025-01921-0 (PMC12269308; doi:10.1186/s13148-025-01921-0)
Supplement: Supplementary file 1 — Additional file 1. [file 13148_2025_1921_MOESM1_ESM.pdf]

Supplementary data

Martinostat as a Novel HDAC Inhibitor to Overcome Tyrosine Kinase Inhibitor Resistance in Chronic Myeloid Leukemia

Haeun Yang[1], Vladimir Li[1], Su Jung Park[1], Sang Won Cheon[1], Anne Lorant[2], Aloran Mazumder [1,&], Jin Young Lee[1,+], Barbora Orlikova-Boyer[2], Claudia Cerella[2], Christo Christov[3], Gilbert Kirsch[4], Dag Erlend Olberg[5], Guy Bormans[6], Hyoung Jin Kang[7], Byung Woo Han[1], Michael Schnekenburger[2], Marc Diederich[1,*]

[1] Research Institute of Pharmaceutical Sciences & Natural Products Research Institute, College of Pharmacy, Seoul National University, Seoul 08826, Republic of Korea

[2] Laboratoire de Biologie Moléculaire et Cellulaire de Cancer, L-1210, Luxembourg

[3] Service d'Histologie, Faculté de Médicine, Université de Lorraine, INSERM U1256 NGERE, 54000, Nancy, France

[4] UMR CNRS 7053 LC2M, University of Lorraine, 57070, Metz, France

[5] Norsk Medisinsk Syklotronsenter AS, Postboks 4950, 0424 Nydalen, Oslo, Norway and School of Pharmacy, University of Oslo, Oslo, Norway

[6] Laboratory for Radiopharmaceutical Research, Department of Pharmaceutical and Pharmacological Sciences, KU Leuven, Leuven, Belgium

[7] Department of Pediatrics, Seoul National University College of Medicine, Seoul National University Cancer Research Institute, Seoul National University Children's Hospital, Seoul 03080, Republic of Korea.

Present address:

[&] Aging and Cancer Immuno-Oncology, Sanford Burnham Prebys Medical Discovery Institute, La Jolla, CA 92037, USA.

[+] Department of Biotechnology, Keimyung University, 1095 Dalgubeol-daero, Dalseo-gu, Daegu 42601, Republic of Korea

* Corresponding author

Supplementary Materials and Methods

Quantification of Immunohistochemistry Images

Six tumor images were analyzed for each sample. Each 50 µm scale bar image was split in half, and the region of interest was reduced for automated processing. Positive cell quantification employed a multistep segmentation procedure using supervised machine learning based on 37 color, texture, and edge parameters, with ~100 image filters enhancing segmentation accuracy. Segmented images were labeled "export," while binary masks (labeled "Mask") excluded non-relevant pixels, such as debris. The tumor tissue areas were measured at 0.39 µm per pixel. In Ki67 vehicle images, closely adjacent nuclei were separated using a watershed algorithm, which calculates a distance map from each object's center to its edge, forming dams between "watersheds" to distinguish touching objects with roundness ratios (4πarea/perimeter^2) greater than 0.8, typical of labeled nuclei. Quantitative results were expressed as signal density per $1 \times 10^{\wedge 5}$ µm^2 across samples.

Supplementary Tables

Supplementary Table 1: CML patient characteristics.

Patient characteristics	Patient 1	Patient 2
Diagnosis	CML, BC	CML, BC
Gender	Male	Male
Age	20	17
Fish	BCR-ABL1 rearrangement	BCR-ABL1 rearrangement
Cytogenetic abnormalities	46,XY,t(9;22)(q34;q11.2)[23]<br>BCR-ABL major: positive (b2a2 type)	46,XY,t(9;22)(q34;q11.2)[23]<br>BCR-ABL major: positive (b3a2 type)

Abbreviations: BC: blast crisis; BCR-ABL, breakpoint cluster region-Abelson; CML, chronic myeloid leukemia; q, long arm of the chromosome; t, translocation.

Supplementary Table 2: Antibodies used for western blots.

Antibody	Company	Catalog number	Dilution
Acetylated histone H4	Millipore	06-866	1:50000 in 5% skim milk
Acetylated α-tubulin	Santa Cruz	Sc-23950	1:1000 in 5% skim milk
c-ABL	Santa Cruz	Sc-23	1:1000 in 5% skim milk
Caspase-3	Santa Cruz	Sc-56053	1:1000 in 5% skim milk
Caspase-7	Cell Signaling	9494	1:1000 in 5% skim milk
Caspase-8	Cell Signaling	9746	1:1000 in 5% BSA
Caspase-9	Cell Signaling	9502	1:1000 in 5% skim milk
Histone H1	Santa Cruz	Sc-8030	1:2000 in 5% BSA
Histone H4	Cell Signaling	Sc-2592	1:2000 in 5% BSA
PARP-1	Santa Cruz	Sc-53643	1:1000 in 5% skim milk
P-BCR (Tyr177)	Cell Signaling	3901S	1:1000 in 5% BSA
P-STAT5	Cell Signaling	9351	1:1000 in 5% skim milk
STAT5	Cell Signaling	94205	1:1000 in 5% BSA
α-tubulin	Sigma-Aldrich	CP06	1:2000 in 5% skim milk
β-actin	Sigma-Aldrich	A5441	1:20000 in 5% skim milk

ABL: Abelson, BCR: breakpoint cluster region, BSA: bovine serum albumin, P: phosphorylated, PARP1: poly (ADP-ribose) polymerase 1, STAT5: signal transducer and activator of transcription 5.

Supplementary Table 3: Combination index for martinostat and imatinib treatments in chronic myeloid leukemia cell lines.

Cell line	Martinostat (µM)	Imatinib (µM)	Combination index
**K562**	0.15	0.1	0.48
	0.15	0.15	0.52
	0.25	0.1	0.53
	0.25	0.15	0.59
**K562-R**	0.15	0.5	0.34
	0.15	1	0.47
	0.25	0.5	0.49
	0.25	1	0.58

Supplementary figure legends

Fig. S1. Synthesis scheme for the histone deacetylase inhibitor martinostat.

Fig. S2. Docking studies of martinostat and SAHA against HDAC isoenzymes.
(**A**) Comparison of the overall structure and catalytic domain of HDAC isoenzymes through overlapping docking results using martinostat. (**B**) Docking simulation results of martinostat and suberoylanilide hydroxamic acid (SAHA) on the crystal structures of histone deacetylase (HDAC) isoenzymes (PDB ID: 4BKX, 4LXZ, 4A69, 2VGM, 5EDU, 3C0Z, 1T69, and 7SGG for HDAC1, HDAC2, HDAC3, HDAC4, HDAC6, HDAC7, HDAC8 and HDAC10, respectively). During docking simulations and binding affinity predictions using MM/GBSA calculations, the Zn^{2+} ion (gray beads) was retained without modification due to its critical role in ligand binding. Additionally, K^+ and Na^+, SO_4^+ (purple, green, and red beads, respectively), were preserved to maintain the protein's structural stability, minimizing variations that could impact binding results.

Fig. S3. Immunoblot analyses and quantification of α-tubulin and histone H4 expression in K562 cells.
Upper panel: three independent blot images of α-tubulin and histone H4 in K562 cells after treatment with martinostat and SAHA at indicated concentrations for 24 hours. β-actin was a loading control for α-tubulin, and histone H1 was a loading control for histone H4. Lower panel: quantifications of western blot signals. Data are the mean ± SD of three independent experiments. Statistical significance was assessed using one-way ANOVA with Dunnett's multiple comparison test, versus control cells.

Fig. S4. Anti-leukemic properties of martinostat and SAHA in imatinib-sensitive and resistant CML cells.
Quantification of the total area and average size of colonies in imatinib-sensitive and -resistant CML cells after treatment with increasing concentrations of martinostat or SAHA for 10 days. The colony-forming capacity was scored after the addition of MTT. Data are presented as the mean ± SD of three independent experiments. P-values were measured using one-way ANOVA with Dunnett's multiple comparison test. *, ** and *** indicate $P < 0.05$, $P < 0.01$ and $P < 0.001$, respectively, versus control cells. A.u.: arbitrary units.

Fig. S5. Computational analysis of transcriptomic data from martinostat-treated CML cells.

K562 cells were treated with 0, 0.15, 0.25, 0.5, and 1 μM martinostat for 24h and analyzed by mRNA sequencing. **(A)** Volcano plots of the martinostat-treated groups compared with the control. Volcano plots show differentially expressed genes with $\log_2$ fold change > 1 and an adjusted P-value < 0.05. Genes identified as upregulated are represented in red, whereas those identified as downregulated are shown in blue. **(B)** Heat maps of martinostat-treated groups. The expression patterns of 121 genes across three categories (epigenetic chromatin modification enzymes, DNA repair, and apoptotic processes) are shown and classified based on z-score values. The heatmap illustrates the mRNA levels differentially expressed at the indicated concentrations of martinostat.

Fig. S6. mRNA-sequencing analysis of selected cell cycle-related genes in martinostat-treated K562 cells.

K562 cells were treated with 0, 0.15, 0.25, 0.5, and 1 μM martinostat for 24h and analyzed by mRNA sequencing. Gene expression levels were compared based on normalized fragments per kilobase of transcript per million mapped reads (FPKM). Data are presented as the mean ± SD of three independent experiments. P-values were measured using one-way ANOVA with Dunnett's multiple comparison test. *, ** and *** indicate P < 0.05, P < 0.01 and P < 0.001, respectively, versus control cells. CCN, cyclin; CDKN, cyclin-dependent kinase; E2F, E2F transcription factor.

Fig. S7. HMGB1 release after martinostat treatment in imatinib-sensitive and -resistant CML cells.

Imatinib-sensitive and -resistant CML cells were treated with martinostat at the indicated concentrations for 24 h, and high mobility group box 1 (HMGB1) release was measured and compared to imatinib. Data are presented as the mean ± SD of three independent experiments. P-values were measured using one-way ANOVA with Dunnett's multiple comparison test. * indicates P < 0.05 versus control cells.

Fig. S8. mRNA-sequencing analysis of transcriptomic data of autophagy-related genes in martinostat-treated K562 cells.

K562 cells were treated with 0, 0.15, 0.25, 0.5, and 1 μM martinostat for 24h and analyzed by mRNA sequencing. Heatmaps of martinostat-treated groups showed the expression patterns of 40 genes involved in the autophagy pathway, which were classified based on z-score values. The heatmap illustrates the mRNA levels differentially expressed at the indicated concentrations of martinostat.

Fig. S9. Electron microscopy observation of martinostat-treated imatinib-sensitive and

resistant CML cells.

Representative transmission electron microscopy images of K562-R, KBM5, and KBM5-IR cells treated for 24 h.

Fig. S10. Colony formation assay in KBM5 and MEG01 CML cells after treatment with martinostat alone or combined with imatinib.

KBM5 and MEG01 cells were treated with the indicated concentrations of martinostat or imatinib. After 10 days of incubation, the colony-forming capacity was scored following the addition of MTT. Upper panel: Representative images of three independent assays and their corresponding quantification (lower panel). P-values were measured using one-way ANOVA with Šídák's multiple comparison test. *, ** and *** indicate $P < 0.05$, $P < 0.01$ and $P < 0.001$, respectively, versus control. ## and ### indicate $P < 0.01$ and $P < 0.001$, respectively, versus combination. A.u.: arbitrary units.

Fig. S11. Differential gene expression analysis of K562-R cells compared to K562 cells.

(A) Volcano plots of K562-R cells compared with K562 cells. Volcano plots show differentially expressed genes with $\log_2$ fold change > 2 and an adjusted P-value < 0.05. Genes identified as upregulated are represented in red, whereas those identified as downregulated are shown in blue. **(B)** Heatmap of complete gene expression patterns for K562 and K562-R cells. The heatmap illustrates the mRNA levels differentially expressed for the genes in the triplets.

Fig. S12. Top 20 Gene Ontology (GO) terms for upregulated and downregulated differentially expressed genes (DEGs).

(A) The top 20 significantly (P-values < 0.05) enriched categories of upregulated genes. **(B)** The top 20 significantly (P-values < 0.05) enriched categories of downregulated genes

Fig. S13. Computational analysis of transcriptomic data from martinostat-treated CML cells.

K562 cells were treated with 0, 0.15, 0.25, 0.5, and 1 μM martinostat for 24h and analyzed by mRNA sequencing. Heatmaps of the martinostat-treated groups are presented. The expression patterns of the 26 genes involved in the BCR-ABL-STAT5 pathway category are shown and classified based on z-score values. The heatmap illustrates the mRNA levels differentially expressed at the indicated concentrations of martinostat.

Fig. S14. Tumor volume measurement of BALB/c-nude mice xenografted with K562-R tumor cells and treated with martinostat alone or combined with imatinib.

The tumor volume of mice ($n = 3$ per group) treated with martinostat alone or combined with imatinib was measured over 18 days of the experiment. Data are the mean ± SD. P-values were analyzed using one-way ANOVA with Dunnett's multiple comparison test. ** and *** indicate $P < 0.01$ and $P < 0.001$, respectively, versus vehicle control.

Fig. S15. Quantification of the histological analysis of K562-R tumor xenografts in BALB/c-nude mice treated with martinostat alone or combined with imatinib.

Immunochemistry image quantification was conducted using the original region of interest images of all IHCs. Data are presented as the mean ± SD of three independent experiments. P-values were measured using one-way ANOVA with Šídák's multiple comparison test. *** indicate $P < 0.001$, versus vehicle control. ## and ### indicate $P < 0.01$ and $P < 0.001$, respectively, versus combination.
