# Supplementary material for: Martinostat as a novel HDAC inhibitor to overcome tyrosine kinase inhibitor resistance in chronic myeloid leukemia
Source: Clin Epigenetics. 2025 Jul 16;17:125. doi: 10.1186/s13148-025-01921-0 (PMC12269308; doi:10.1186/s13148-025-01921-0)
Supplement: Supplementary file 3 — Additional file 3. [file 13148_2025_1921_MOESM3_ESM.pdf]

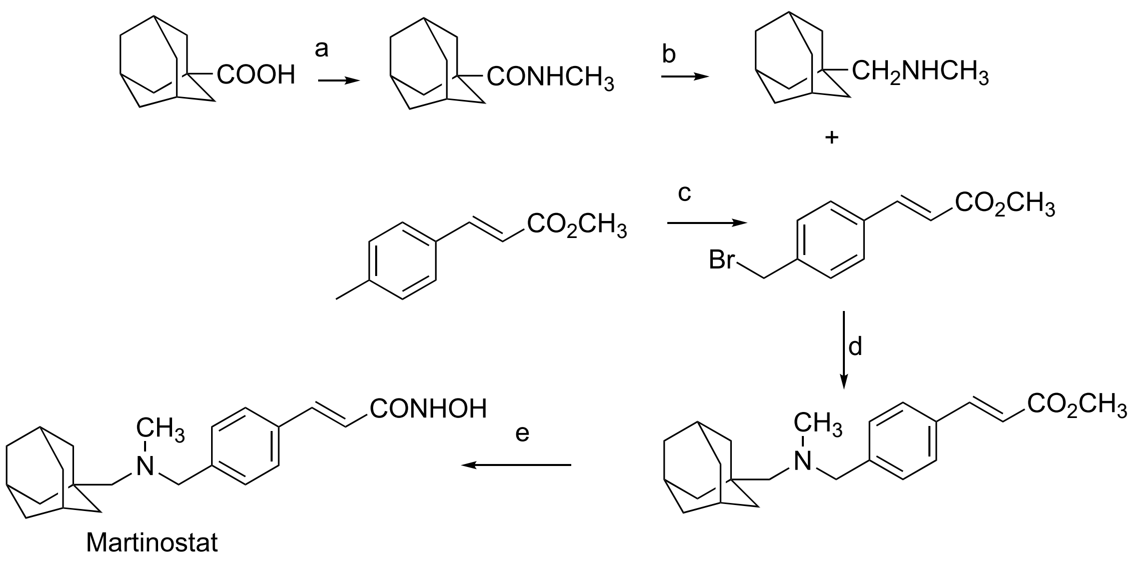

a) $SOCl_2$ $CHCl_3$; CH_3NH_2 50% in water, 80%
b) $LiAlH_4$, THF, 70%
c) NBS, chlorobenzene, reflux
d) K_2CO_3, CH_3CN, overnight, 60%
e) NH_2OH aq, 1M NaOH, MeOH-THF, 0°C to RT, 12h. 30%

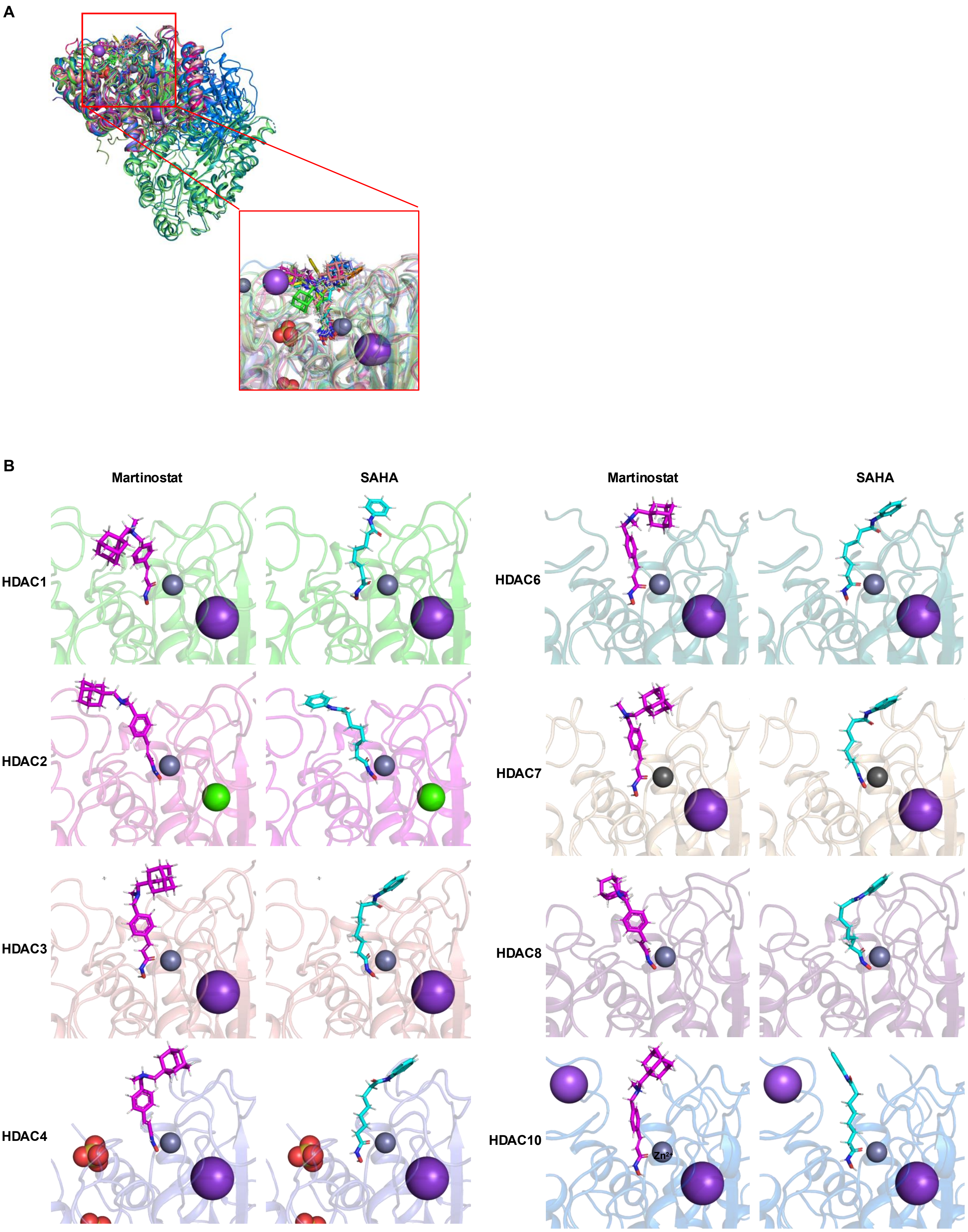
A
B
Martinostat
SAHA
Martinostat
SAHA
HDAC1
HDAC2
HDAC3
HDAC4
HDAC6
HDAC7
HDAC8
HDAC10
Zn2+

Figure S3, Yang *et al.*

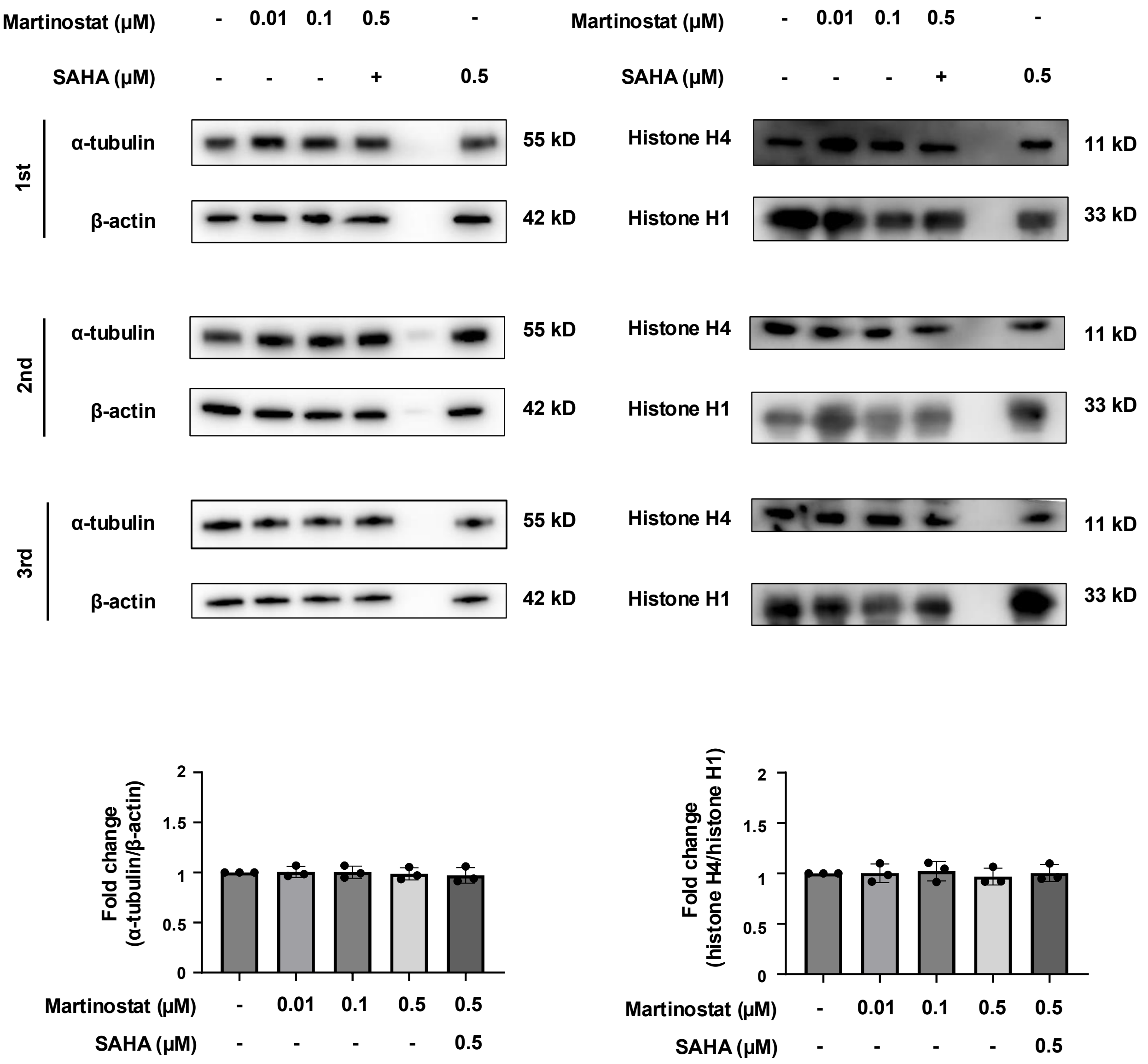

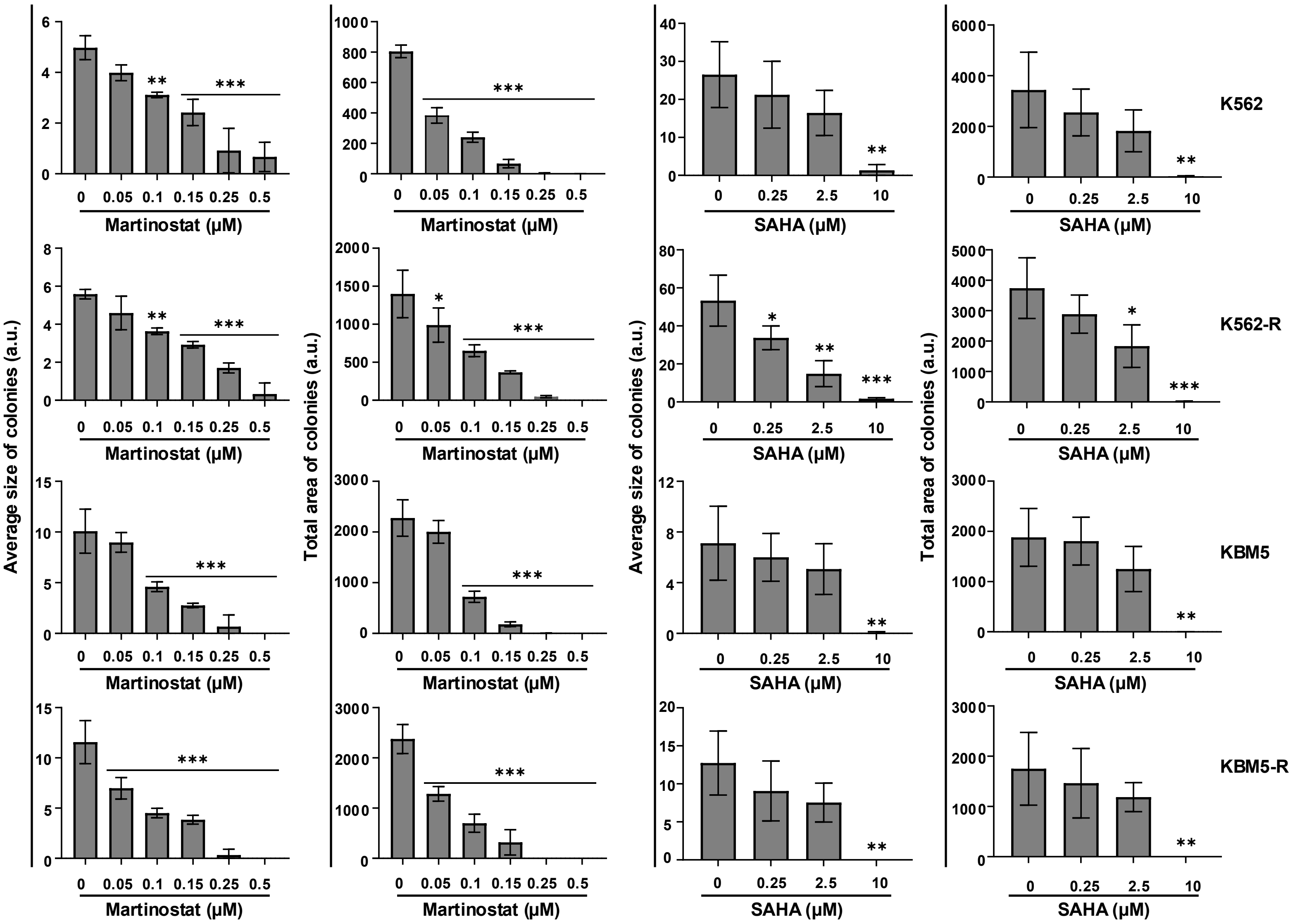
Average size of colonies (a.u.)
Total area of colonies (a.u.)
Martinostat (μM)
SAHA (μM)
0
0.05
0.1
0.15
0.25
0.5
2.5
10
K562
K562-R
KBM5
KBM5-R

A

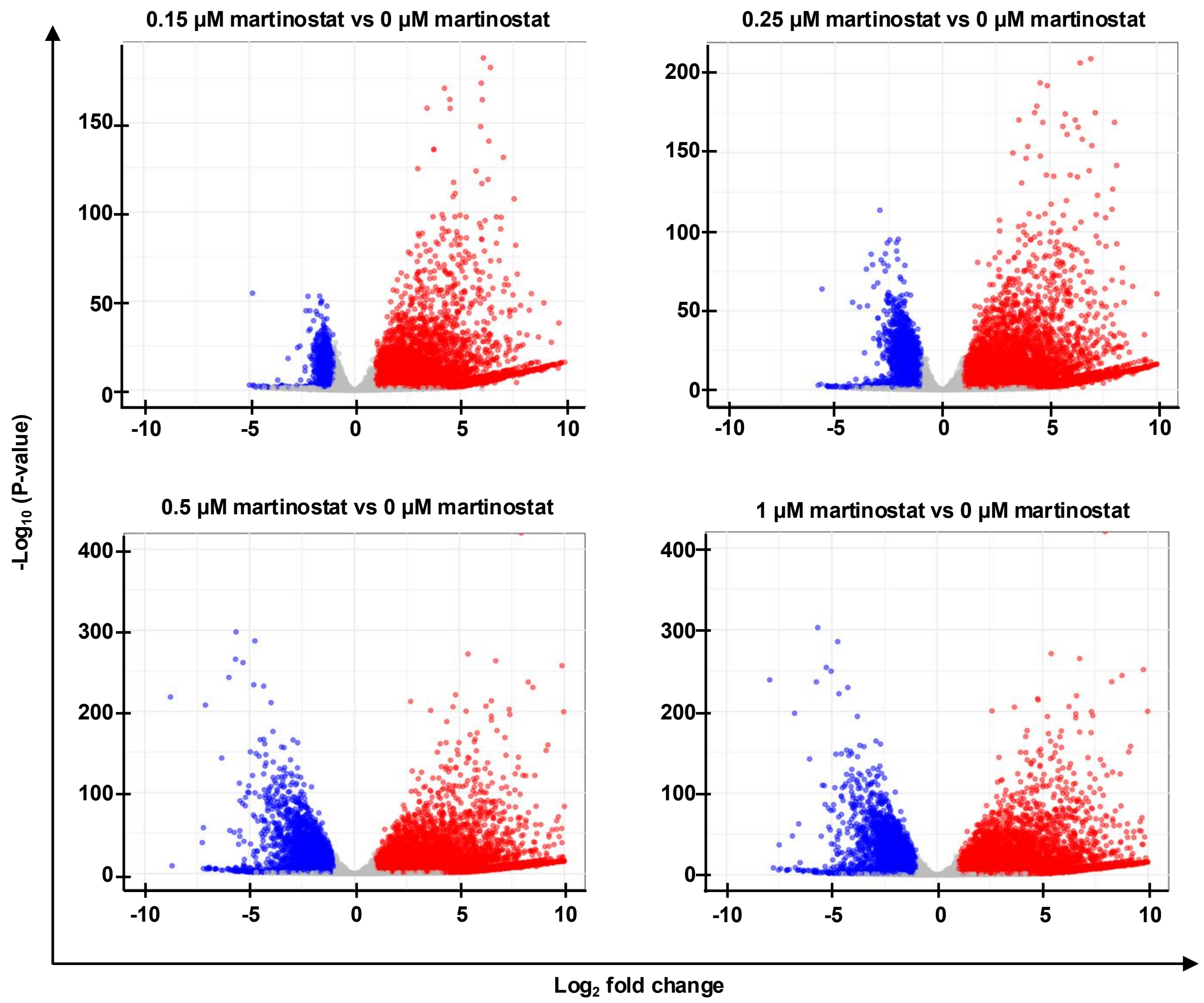
0.15 µM martinostat vs 0 µM martinostat
0.25 µM martinostat vs 0 µM martinostat
0.5 µM martinostat vs 0 µM martinostat
1 µM martinostat vs 0 µM martinostat
-Log10 (P-value)
Log2 fold change

B

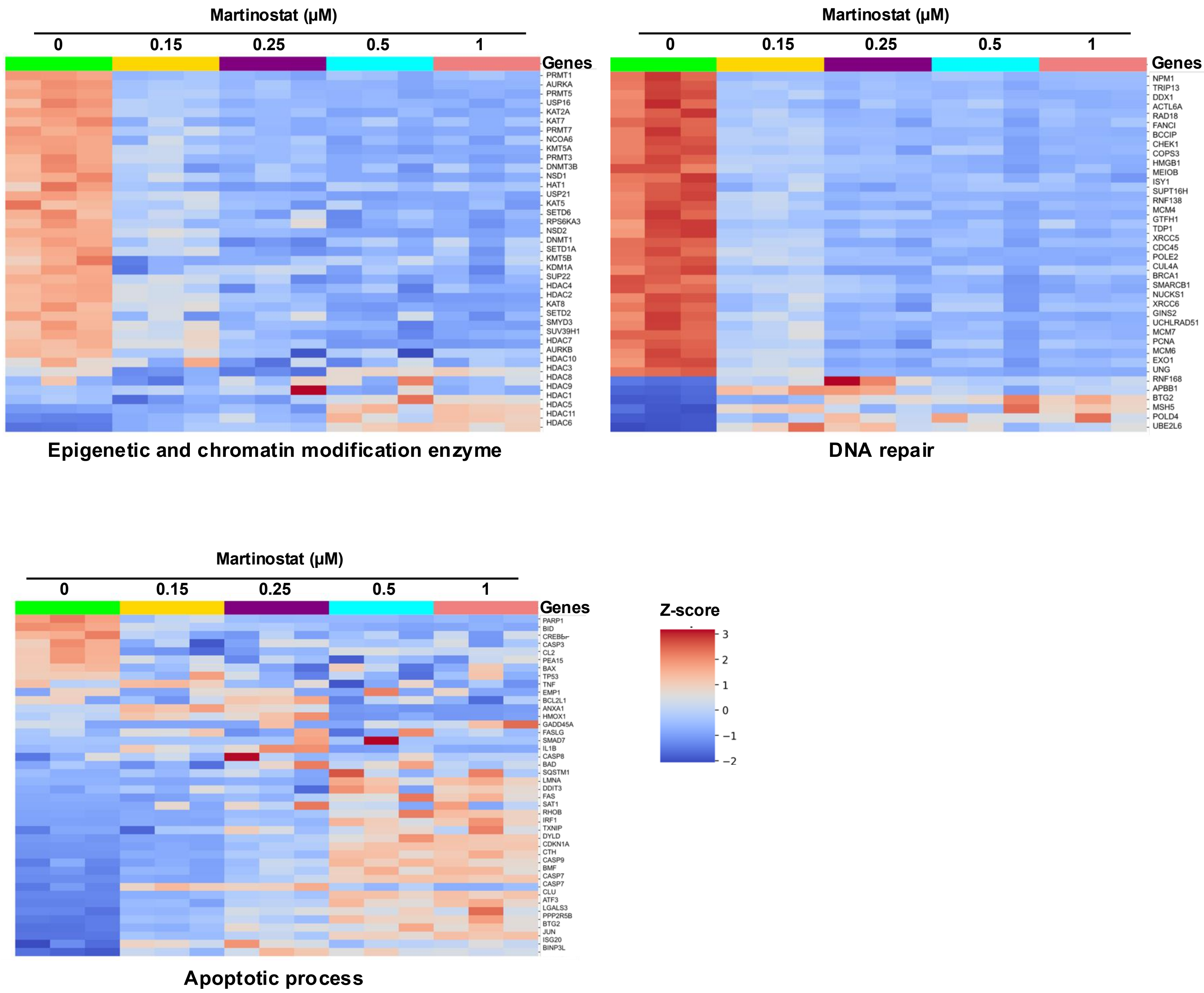
Martinostat (µM)
0
0.15
0.25
0.5
1
Genes
PRMT1
AURKA
PRMT5
USP16
KAT2A
KAT7
PRMT7
NCOA6
KMT5A
PRMT3
DNMT3B
NSD1
HAT1
USP21
KAT5
SETD6
RPS6KA3
NSD2
DNMT1
SETD1A
KMT5B
KDM1A
SUP22
HDAC4
HDAC2
KAT8
SETD2
SMYD3
SUV39H1
HDAC7
AURKB
HDAC10
HDAC3
HDAC8
HDAC9
HDAC1
HDAC5
HDAC11
HDAC6
Epigenetic and chromatin modification enzyme
NPM1
TRIP13
DDX1
ACTL6A
RAD18
FANCI
BCCIP
CHEK1
COPS3
HMGB1
MEIOB
ISY1
SUPT16H
RNF138
MCM4
GTFH1
TDP1
XRCC5
CDC45
POLE2
CUL4A
BRCA1
SMARCB1
NUCKS1
XRCC6
GINS2
UCHLRAD51
MCM7
PCNA
MCM6
EXO1
UNG
RNF168
APBB1
BTG2
MSH5
POLD4
UBE2L6
DNA repair
PARP1
BID
CREBBP
CASP3
CL2
PEA15
BAX
TP53
TNF
EMP1
BCL2L1
ANXA1
HMOX1
GADD45A
FASLG
SMAD7
IL1B
CASP8
BAD
SQSTM1
LMNA
DDIT3
FAS
SAT1
RHOB
IRF1
TXNIP
DYLD
CDKN1A
CTH
CASP9
BMF
CASP7
CASP7
CLU
ATF3
LGALS3
PPP2R5B
BTG2
JUN
ISG20
BINP3L
Apoptotic process
Z-score

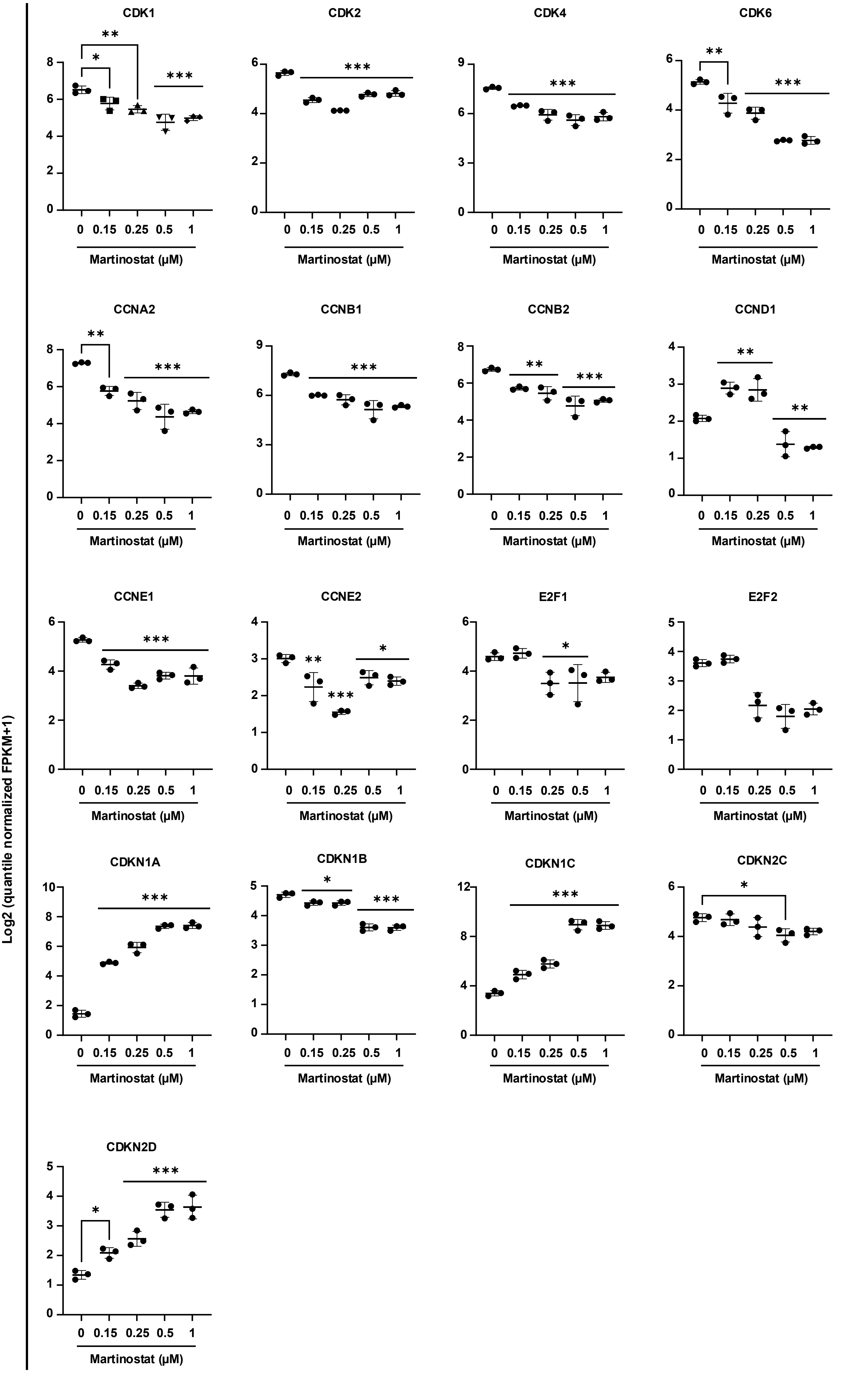
Log2 (quantile normalized FPKM+1)
CDK1
CDK2
CDK4
CDK6
CCNA2
CCNB1
CCNB2
CCND1
CCNE1
CCNE2
E2F1
E2F2
CDKN1A
CDKN1B
CDKN1C
CDKN2C
CDKN2D
0 0.15 0.25 0.5 1
Martinostat (μM)

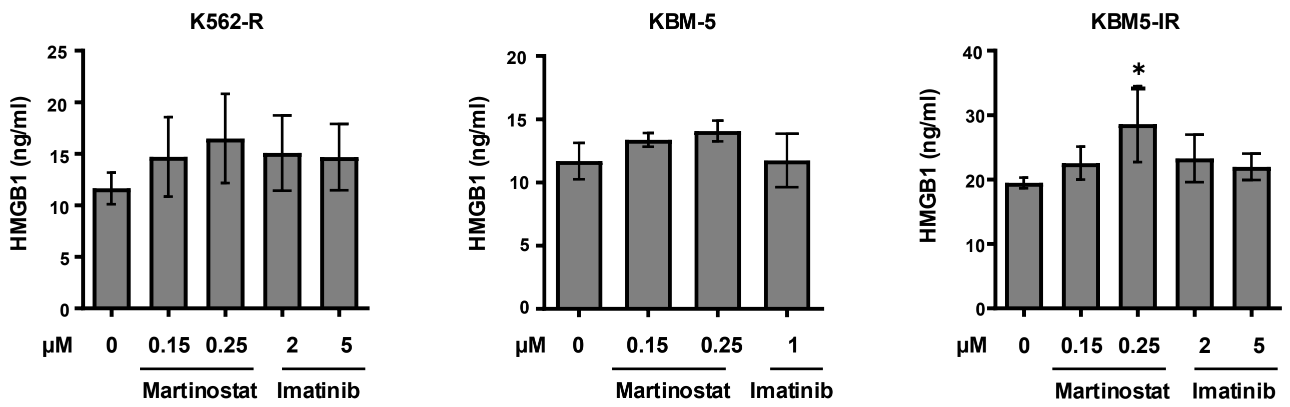
K562-R
HMGB1 (ng/ml)
25
20
15
10
5
0
μM
0
0.15
0.25
2
5
Martinostat
Imatinib
KBM-5
HMGB1 (ng/ml)
20
15
10
5
0
μM
0
0.15
0.25
1
Martinostat
Imatinib
KBM5-IR
HMGB1 (ng/ml)
40
30
20
10
0
*
μM
0
0.15
0.25
2
5
Martinostat
Imatinib

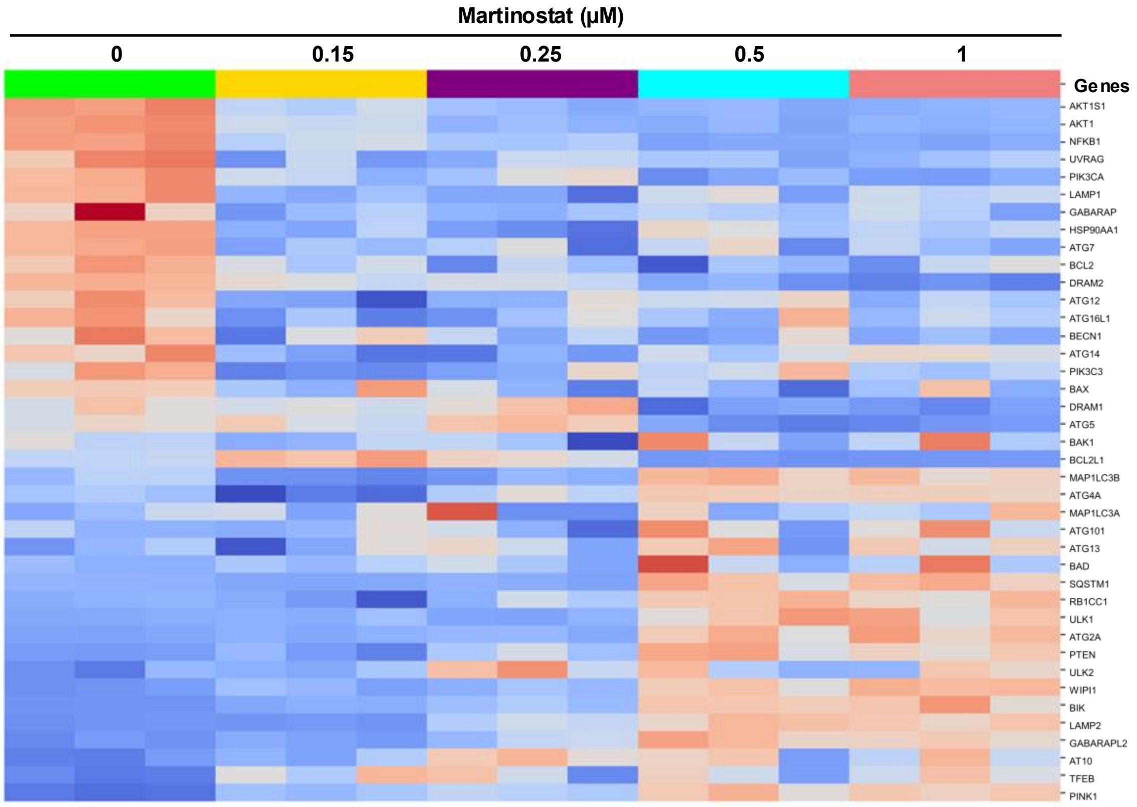
Martinostat (μM)
0
0.15
0.25
0.5
1
Genes
AKT1S1
AKT1
NFKB1
UVRAG
PIK3CA
LAMP1
GABARAP
HSP90AA1
ATG7
BCL2
DRAM2
ATG12
ATG16L1
BECN1
ATG14
PIK3C3
BAX
DRAM1
ATG5
BAK1
BCL2L1
MAP1LC3B
ATG4A
MAP1LC3A
ATG101
ATG13
BAD
SQSTM1
RB1CC1
ULK1
ATG2A
PTEN
ULK2
WIPI1
BIK
LAMP2
GABARAPL2
AT10
TFEB
PINK1

Autophagy

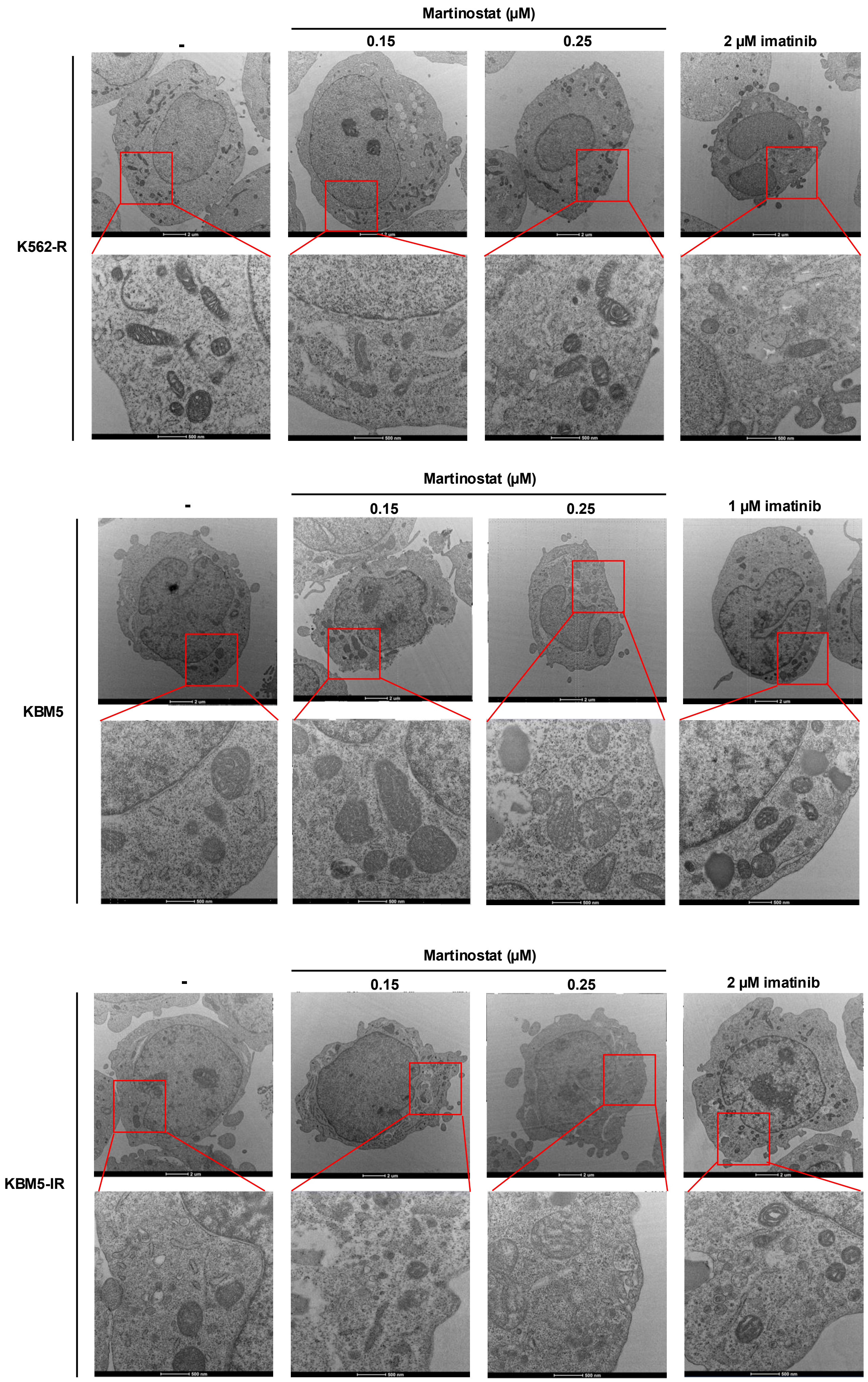
Martinostat (μM)
-
0.15
0.25
2 μM imatinib
K562-R
Martinostat (μM)
-
0.15
0.25
1 μM imatinib
KBM5
Martinostat (μM)
-
0.15
0.25
2 μM imatinib
KBM5-IR

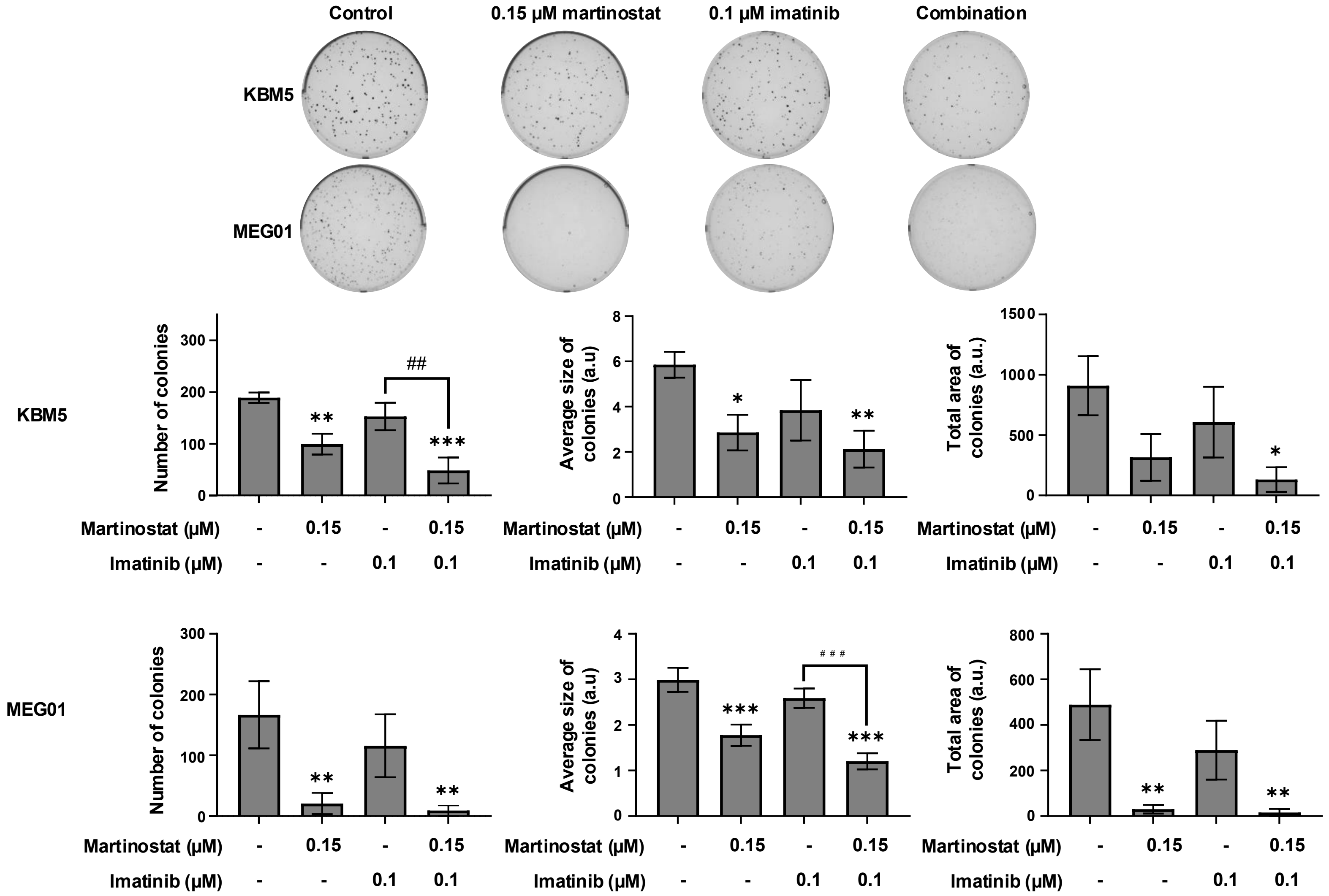

Control
0.15 μM martinostat
0.1 μM imatinib
Combination
KBM5
MEG01
Number of colonies
Average size of colonies (a.u)
Total area of colonies (a.u.)
##
###
Martinostat (μM)
Imatinib (μM)
0.15
0.1

A

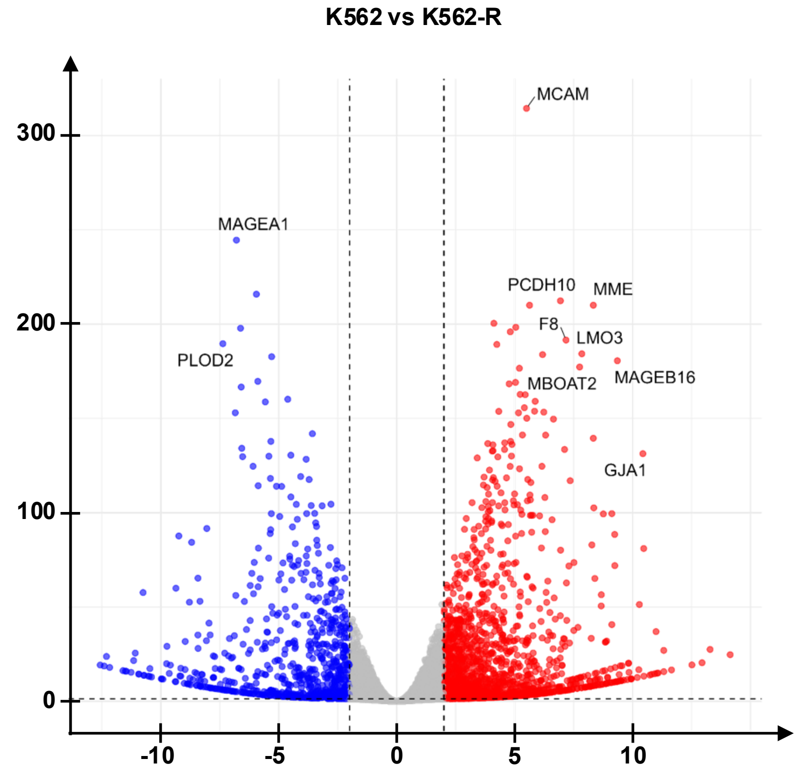
K562 vs K562-R
MCAM
MAGEA1
PLOD2
PCDH10
MME
F8
LMO3
MAGEB16
MBOAT2
GJA1
300
200
100
0
-10
-5
0
5
10

B

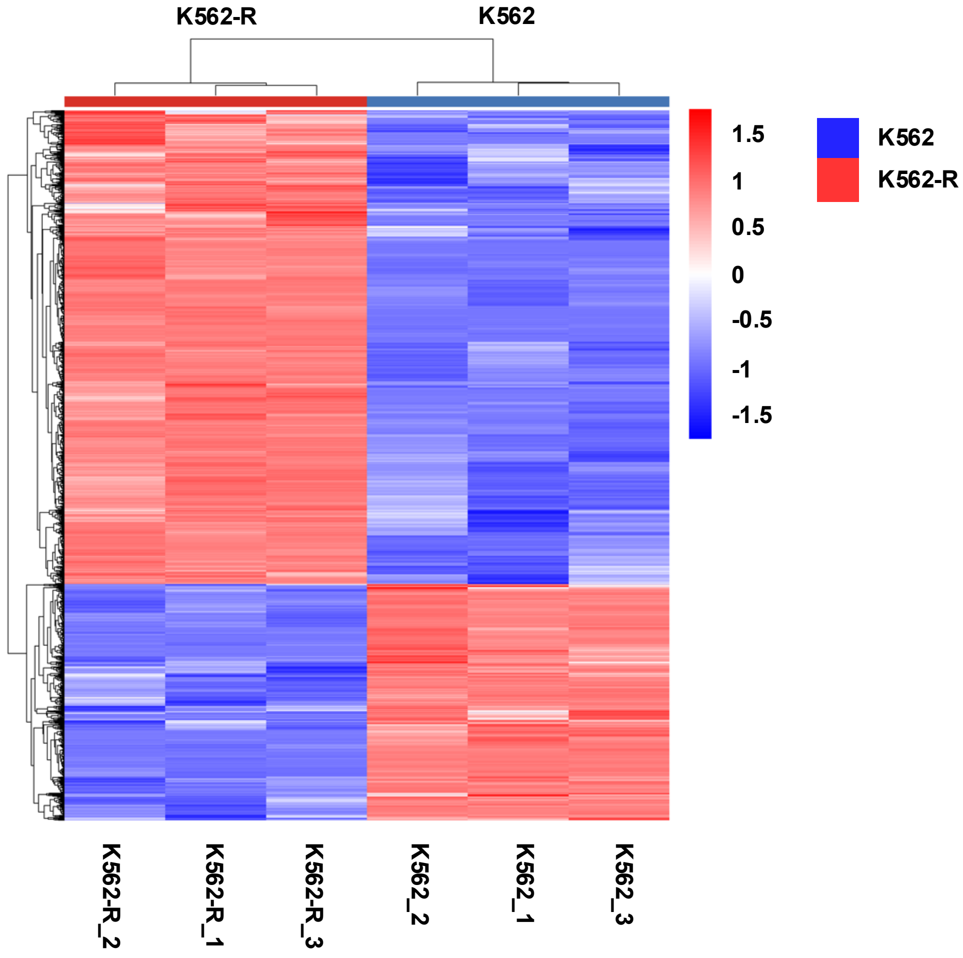
K562-R
K562
1.5
1
0.5
0
-0.5
-1
-1.5
K562
K562-R
K562-R_2
K562-R_1
K562-R_3
K562_2
K562_1
K562_3

Figure S12, Yang *et al.*

A

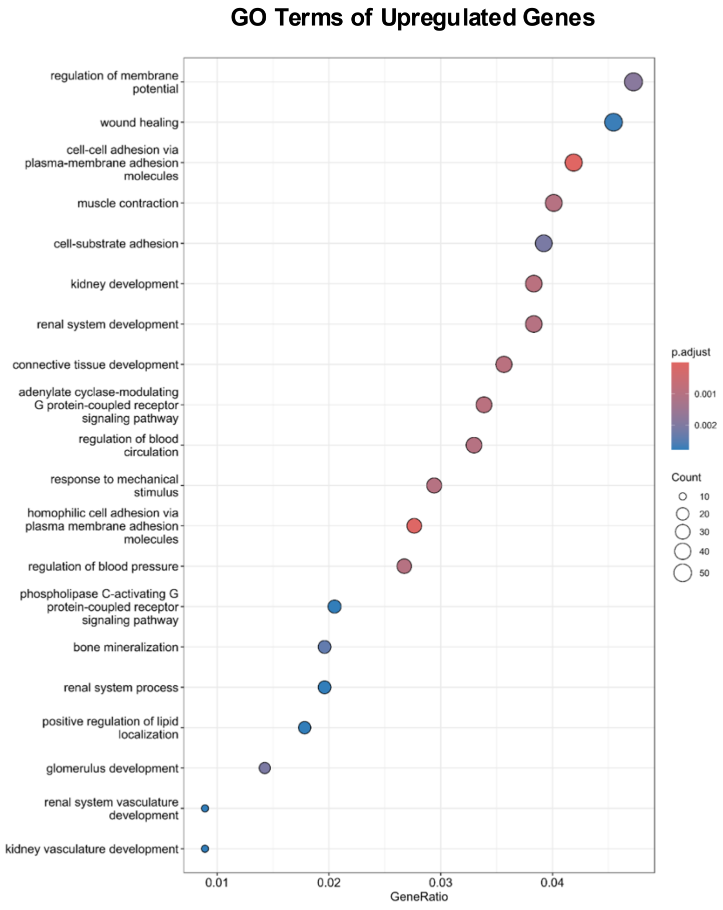

B

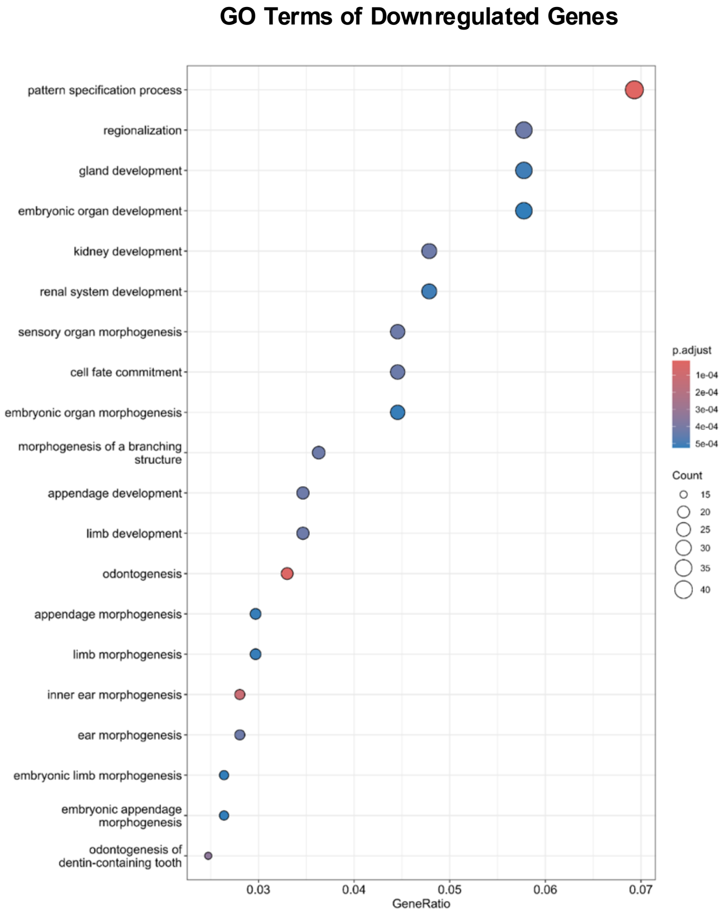

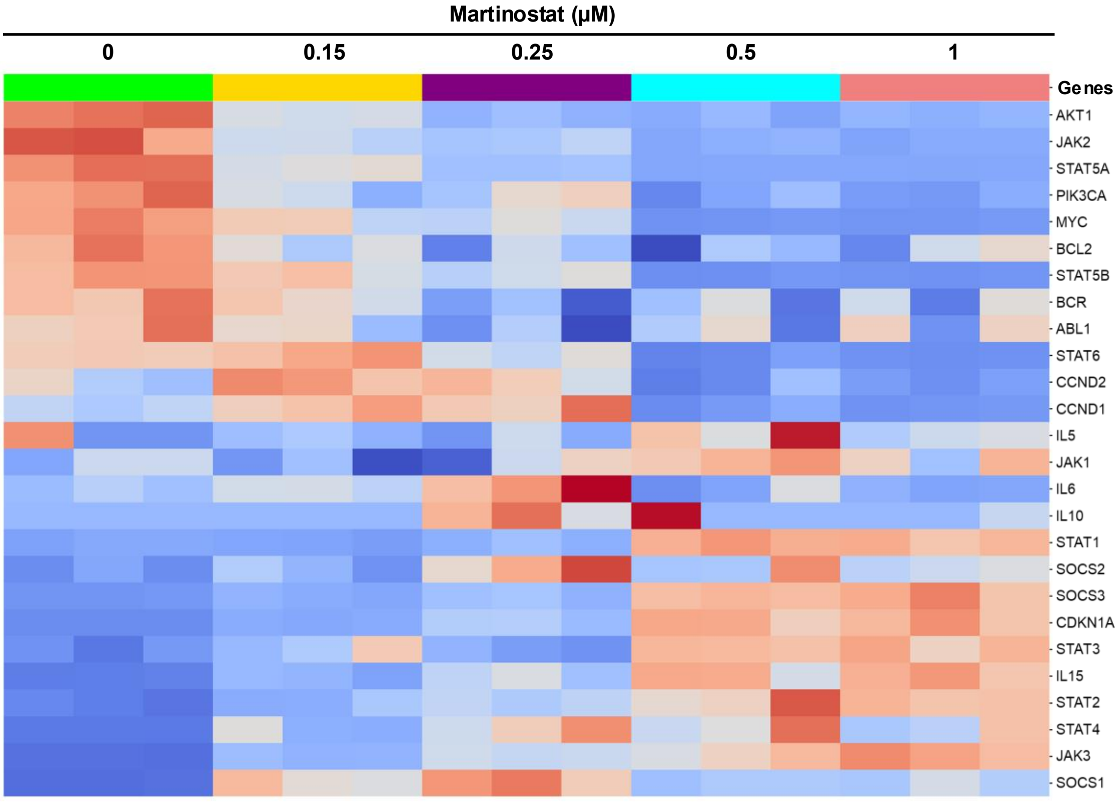

BCR-ABL – STAT5 pathway

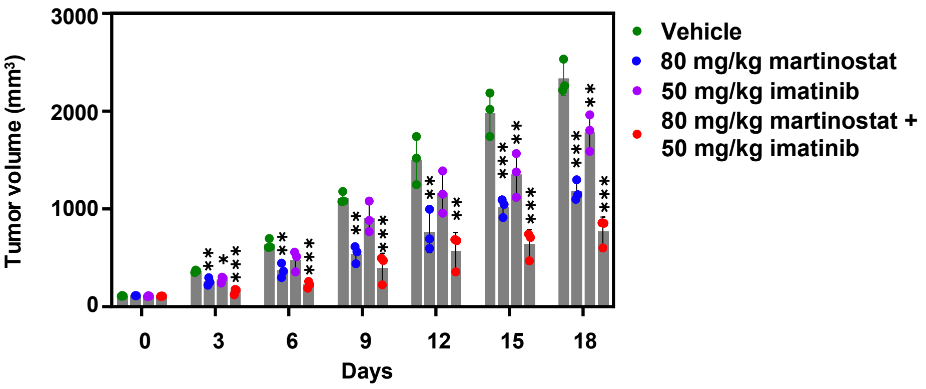

3000
2000
1000
0
Tumor volume (mm³)
0
3
6
9
12
15
18
Days
Vehicle
80 mg/kg martinostat
50 mg/kg imatinib
80 mg/kg martinostat +
50 mg/kg imatinib

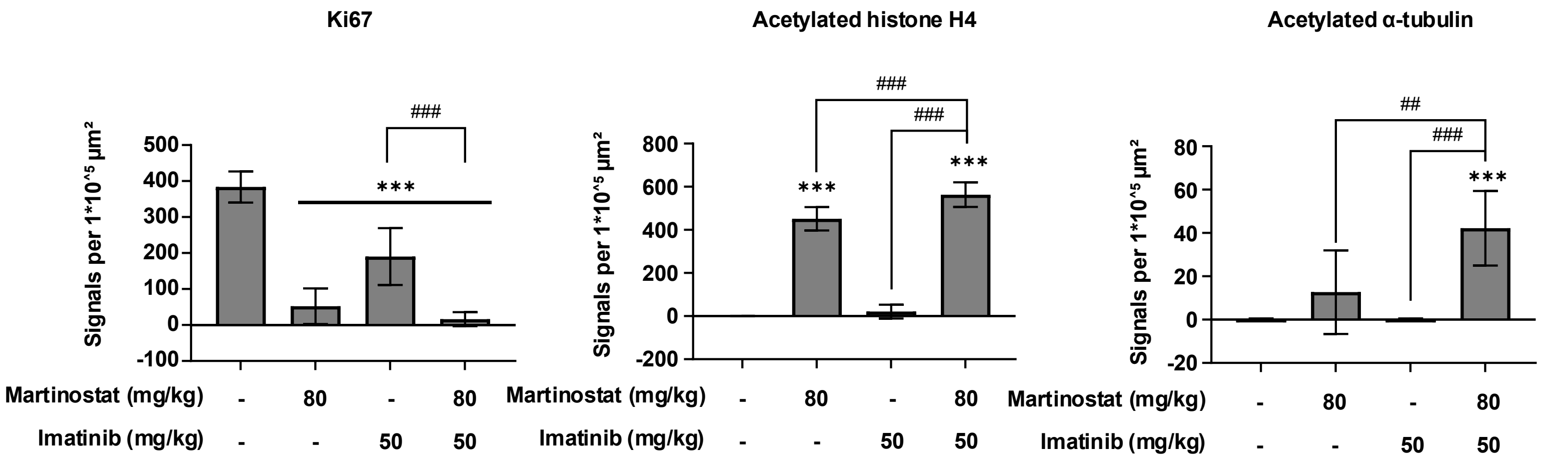
Ki67
Acetylated histone H4
Acetylated α-tubulin
Signals per 1*10^5 µm²
***
###
##
Martinostat (mg/kg)
Imatinib (mg/kg)
- 80 - 80
- - 50 50
